# Supplementary material for: Integration of serial self-testing for COVID-19 as part of contact tracing in the Brazilian public health system: A pragmatic trial protocol
Source: PLoS One. 2023 Oct 4;18(10):e0284659. doi: 10.1371/journal.pone.0284659 (PMC10550143; doi:10.1371/journal.pone.0284659)
Supplement: S1 File — (PDF) [file pone.0284659.s001.pdf]

Integration of Serial Self-Testing into Public Health Contact Tracing Programs: A Pragmatic Trial to Assess the Operational Feasibility and Impact of COVID-19 Self-Testing among Exposed Individuals in Brazil

Sponsor: PATH
Version: 1.0
Date: 31 August 2022

Table of Contents

List of Abbreviations 3
Key Roles and Contact Information 5
Protocol Summary 7
1.0 Background and rationale for the study 11
2.0 Study objectives and endpoints 14
3.0 Study design 15
3.1 Overall design 16
3.2 Study Site 20
3.3 Qualitative and usability data collection **Error! Bookmark not defined.**
4.0 Current practices and study interventions 21
4.1 Diagnosis and case management standard of care 21
4.2 Arm 1: Control Arm contact tracing 23
4.3 Arm 2: Study intervention 23
5.0 Research participants 25
5.1 Index Cases 25
5.2 Primary Exposures 25
5.3 Usability workshop participants 26
5.4 Focus group participants 26
6.0 Study procedures 26
6.1 Index Cases 28
6.2 Primary Exposures/Close contacts 28
6.3 Test result return and follow-up 30
6.4 Secondary Exposures 30
6.5 Usability 30
6.6 Focus Groups 31
7.0 Consent process 31
8.1 Secondary Exposures 32
8.2 Usability Workshop and Focus Groups 32
8.3 Consent to Share GPS Location Data 33
9.0 Statistical Considerations 33
9.1 Overview and General Design 33
9.2 Sample Size: Effectiveness 33
9.3 Analytical methods: Effectiveness and operational feasibility 34
9.4 Concordance of Self-Tests with Professionally Administered Ag-RDTs 34
9.5 Sample Size and Analytical Methodology: Usability Study 34
9.6 Qualitative analysis of intervention barriers and facilitators 34

9.7 Documentation of commodity costs for self-testing supported contact tracing 35
10.0 Confidentiality and data management 35
10.1 Data collection, entry, and quality control 35
10.2 Data Access and Storage 35
10.3 GPS Data 36
11.0 Study and safety monitoring 36
11.1 Care for injury 37
12.0 Ethical Considerations 37
12.1 Risks and mitigation of risks 37
12.2 Benefits 38
12.3 Study costs and compensation 38
12.4 COVID-19 considerations 38
12.5 Ethical Review 38
12.6 Compliance with Resolutions CNS 466/2012 and 580/2018 39
13.0 Study limitations 40
14.0 Research dissemination and stakeholder engagement 41
15. Investigator responsibilities and implementation 41

List of Abbreviations

ANVISA	Brazilian Health Regulatory Agency (Agência Nacional de Vigilância Sanitária)
CEPEM	Tropical Medicine Research Center (Centro de Pesquisa em Medicina Tropical de Rondônia)
CONEP	The National Commission for Research Ethics (Comissão Nacional de Ética em Pesquisa)
COVID-19	Coronavirus disease
CRF	Case Report Form
EUA	Emergency Use Authorization
FN	False Negative
FP	False Positive
GCP	Good Clinical Practice
ICH	International Conference on Harmonisation
IRB	Institutional Review Board
NAAT	Nucleic acid amplification testing
NPV	Negative Predictive Value
POC	Point-of-Care
PPV	Positive Predictive Value
RDT	Rapid Diagnostic Test
RT-PCR	Reverse transcription polymerase chain reaction
ST	Self-test
TN	True Negative

TP	True Positive
WHO	World Health Organization

Key Roles and Contact Information

**Principal Investigator**	Dhélio Pereira, MD PhD<br>*Clinical Director, Centro de Pesquisa em Medicina Tropical de Rondônia (CEPEM)*<br>*Porto Velho, Rondônia, Brazil*<br>dbpfall@gmail.com
**Co-Investigators**	Camilo Manchola-Castillo, PhD<br>*Programs Manager, Global Health Strategies Brazil*<br>*Rio de Janeiro, RJ, Brazil*<br>cmanchola@globalhealthstrategies.com<br><br>Alexandre Tavares Dias Costa, PhD<br>*Research Scientist, Fiocruz Paraná*<br>*Curitiba, PR, Brazil*<br>alexandre.costa@fiocruz.br<br><br>Michelle Oliveira Silva, PhD<br>*Lab Manager, CEPEM*<br>*Porto Velho, Rondônia, Brazil*<br>oliveirasilvam@gmail.com<br><br>Emily Gerth-Guyette, MPH<br>*Program Officer, PATH*<br>*Seattle, WA, USA*<br>egerthguyette@path.org<br><br>Rebecca Green, MPH<br>*Research Associate, PATH*<br>*Seattle, WA, USA*<br>rgreen@path.org<br><br>Gonzalo Domingo, PhD<br>*Senior Scientific Director, PATH*<br>*Seattle, WA, USA*<br>gdomingo@path.org<br><br>Stephanie Zobrist, MPH<br>*Program Officer, PATH*<br>*Seattle, WA, USA*<br>*szobrist@path.org*<br><br>Kim Green, PhD MA<br>*Global Program Director, Primary Health Care, PATH*<br>*Hanoi, Vietnam*<br>kgreen@path.org<br>*kgreen@path.org*<br><br>Philips Loh

	Regional Technical Officer, HIV/Viral Hepatitis, PATH<br>*Hanoi, Vietnam*<br>*ploh@path.org*
**Ethics Committees**	World Health Organization Ethics Review Committee (WHO ERC)<br>Avenue Appia 20<br>1211 Geneva, Switzerland<br>Tel: 41 22 791 21 11<br><br>Comitê de Ética do Centro de Pesquisa em Medicina Tropical (CEPEM)<br>Avenida Guaporé, 215 Bairro Lagoa- CEP 76812-329<br>Porto Velho, Rondônia, Brazil<br>Tel: 69- 3219-6049, Email: cep.cepem@sesau.ro.gov.br<br><br>Comitê de Ética em Pesquisa da Secretaria Municipal da Saúde<br>Rua Atilio Bório, n° 680, Bairro Cristo Rei, Curitiba<br>CEP 80.050-250<br>Tel: (41) 3360-4961, Email: etica@sms.curitiba.pr.gov.br<br><br>The National Commission for Research Ethics (Comissão Nacional de Ética em Pesquisa [CONEP])<br>SRTV 701, Via W 5 Norte, lote D - Edifício PO 700, 3º andar – Asa Norte<br>Brasília, Brazil<br>Tel: (61) 3315-2951, Fax: (61) 3226-6453, Email: conep@saude.gov.br

Protocol Summary

**Title**	Integration of Serial Self-Testing into Public Health Contact Tracing Programs: A Pragmatic Trial to Assess the Operational Feasibility and Impact of COVID-19 Self-Testing among Exposed Individuals in Brazil
**Short Title**	Evaluation of serial self-testing supported contact tracing for COVID-19 in Brazil
**Protocol Number**	1889025-1
**Précis**	A two-arm randomized pragmatic trial of a COVID-19 contact tracing program with and without serial self-testing. Close contacts of exposed index cases will be identified and contact through a contact-tracing program. They will be randomized to Arm 1 (contact tracing by professional- use rapid diagnostic test at one timepoint) or Arm 2 (serial self-test supported contact tracing). Those who are randomized into Arm 2 will first conduct a supervised self-test to ensure appropriate use of subsequent self-tests. An observational sub-study will be conducted among those with secondary exposures (household members of primary exposure close contacts), who will be offered self-tests for serial use. All testing and self-testing is voluntary. Data will be collected from participants in both arms regarding symptoms, health behaviours, and any test results via digital follow-up surveys. Anonymous data will be submitted by secondary exposure contacts for any of these tests that are used. Focus group discussions and a participatory usability workshop will inform development of additional user resources for serial self-testing as well as to understand perceptions of and experiences with self-testing within contact tracing programmes.
**Study definitions**	• Serial self-testing- For the purposes of the study, participants who have been in contact with a positive index case will be provided 10 Ag-RDT self-tests that they will be asked to used daily for 10 days.<br>• Index case- Individual who has tested positive for COVID-19 either by a laboratory PCR test or an Ag-RDT conducted by a health care provider<br>• Primary exposure: individual who has been exposed to the index case (i.e. within 6 feet of a confirmed positive case for 15 minutes starting 2 days before the onset of the index case symptoms or 2 days before the index case tested positive)<br>• Secondary exposure: individual who shares a household with a primary exposure
**Study Intervention**	The intervention will use SARS-CoV-2 antigen rapid diagnostic tests (Ag-RDT) approved for self-testing by ANVISA in Brazil at the time of study start. Close contacts of confirmed positive cases of COVID-19 will be freely offered up to 10 self-tests and their use will be monitored through remote data collection.
**Study Objectives**<br>**Primary**<br>**Secondary**	<u>Primary</u><br>1.To evaluate the effectiveness of contact tracing supported by serial self-testing (testing daily for up to 10 days) among exposed individuals compared to contact tracing using only professional testing with Ag-RDT performed at one visit<br><br><u>Secondary</u><br>2.To evaluate the operational feasibility of self-testing within the public health contact tracing system

	3.To evaluate the concordance of supervised self-tests as compared to an Ag-RDT conducted by a trained health worker<br>4.To explore barriers and facilitators at the provider and patient levels that mediate use of COVID-19 self-testing and adherence to triage and treatment guidelines<br>5.To generate supportive user resources for self-testing implementation
**Study Endpoints**<br>**Primary**<br>**Secondary**	Primary: Effectiveness<br>1.1 Proportion of primary exposure close contacts who test positive per index case in Arm 2 compared to Arm 1<br>1.2 Proportion of Ag-RDT positive contacts per index case who report adhering to recommended treatment, quarantine, or isolation guidelines at the time study is running in Arm 2 compared to Arm 1<br><br>Secondary: Operational Feasibility of Self-Testing<br>2.1 Proportion of exposed contacts in the self-testing group (Arm 2) who report test results as per local guidelines<br>2.2 Number of self-tests used by individuals with secondary exposures<br>2.3 Proportion of exposed contacts who perform a self-test per manufacturer instructions without critical errors, under supervision<br><br>Secondary: Concordance with Professional Use Ag-RDTs<br>3.1 Concordance of self-test result with professional use Ag-RDT test result<br>Secondary: Implementation<br>4.1 Barriers and facilitators to self-testing: acceptability, preferences, and user perspectives across end users, including patients and health professionals<br><br>Secondary: Supportive Resources<br>5.1 Additional resources adapted with user input for self-test kits
**Study Procedures**	Health units within the municipal health system will be trained on and provided self-tests to use in support of contact tracing. Index cases will be randomized to either standard contact tracing (Arm 1) or contact tracing supported by serial self-testing (Arm 2). Confirmed COVID-19 positive index cases will be recruited through multiple clinical platforms that are normally used by the health system such as eSUS. Close contacts will be identified by index cases via contact elicitation interviews. Eligible primary exposure close contacts will be invited to participate in the study, complete consent and subsequent testing, depending on the Arm to which their index case was randomized.<br><br>Arm 1: Contact Tracing with a Professional Use Ag-RDT at one Timepoint<br>*Day 1:* Participant (close contact) is tested with professional use Ag-RDT and completes baseline study questions.<br>*Day 2-10:* Participant completes study questions (including symptoms, isolation or quarantine behaviors, and any other testing done) through remote means (online or by phone).<br><br>Arm 2: Serial Self-Test Supported Contact Tracing

	Day 1: Participant (close contact) completes supervised self-test, professional use Ag-RDT, and baseline study questions. The order in which samples are collected will alternate based on participant ID to account for sample depletion. Participant is provided with 10 self-tests to use daily over the 10-day follow-up period. This visit may occur at the clinic or the participant's home.<br>*Day 2-10:* Participant uses self-test daily and completes study questions (including submission of ST results, symptoms, and isolation behaviors) through remote means via REDCap electronic data capture system (online or by phone).<br><br><u>Sub-Study 1: Index Cases</u><br>Index cases will be consented and followed for up to 10 days remotely. During the enrollment visit, they will complete a contact elicitation interview and a baseline questionnaire to obtain demographic information and information related to their positive test/symptom onset. They will be asked to submit a daily questionnaire via REDCap electronic data capture system to report information including symptoms and quarantine/isolation behavior.<br><br><u>Sub-Study 2: Secondary Exposure Close Contacts in Arm 2</u><br>Primary exposure close contacts in Arm 2 will be provided up to 3 self-tests for each member of their household secondary exposure close contacts) to use, and anonymous data will be requested for each test used.<br><br><u>Qualitative Study Activities</u><br>A participatory workshop focused on self-test usability and user preferences will be held to gain user insights into questions and mistakes in the workflow and improve the test instructions for use. Participants will be asked about their motivations to report a self-test result and general opinions about self-tests. Focus groups will also be held to gain stakeholder insights into barriers and facilitators for implementing self-testing more broadly in the public health system and other potential use cases.
**Study population**	Participants will be confirmed COVID-19 cases (index cases) and their associated close contacts. Index cases will be 7 years of age or older. Primary exposure close contacts will be enrolled in the main study, including children aged 7 and older. Secondary exposure close contacts (household members of primary exposure close contacts enrolled in Arm 2) will be given self-tests to use in the observational sub-study.<br><br>A subset of patients/primary exposure close contacts, caregivers of primary exposure close contacts, health unit workers, and other key stakeholders will also have the option of participating in a usability workshop and/or focus group discussions. These participants will be aged 18 years and older. Detailed Inclusion/Exclusion criteria for all participant groups can be found in Section 5 and listed below.<br><br><u>Index Cases</u><br>*Inclusion Criteria*<br>- 7 years of age or older

- Positive result for SARS-CoV-2 infection according to an ANVISA-approved Ag-RDT or RT-PCR
- Willing and able to provide informed consent (or assent with parent/legal guardian consent if <18) and comply with study requirements

Exclusion Criteria

- Any study site employees who are involved in the protocol or may have access to study-related data
- Treating clinician deems inappropriate to enroll
- Previous study participant

<u>Primary Exposures</u>

Inclusion Criteria

- 7 years of age or older and meets age indication per the self-test's instructions for use
- Exposure (within 6ft/1 meter for more than 15 minutes, physical contact, or healthcare worker caring for patient without appropriate personal protective equipment) to an enrolled index case in the 2 days prior to symptom onset of the index case or within 7 days of the index case's positive COVID-19 test
- Willing and able to provide informed consent (or assent with parent/legal guardian consent if <18) and comply with study requirements

Exclusion Criteria

- Contraindication to nasal swab
- Any study site employees who are involved in the protocol or may have access to study-related data
- Previous study participant

<u>Usability Workshop Participants</u>

Inclusion Criteria

- 18 years of age or older
- Considered to be an intended user of COVID self-tests
- Willing and able to provide informed consent

Exclusion Criteria

- Study site employee involved with the protocol

<u>Focus Group Participants</u>

Inclusion Criteria

- 18 years of age or older
- Considered to be a relevant stakeholder in future practices or policies around the implementation of STs (e.g., patient, local health department official, healthcare provider, caregiver)
- Willing and able to provide informed consent

Exclusion Criteria

- Study site employee involved with the protocol

**Sample Size**	To demonstrate serial self-testing can identify more positive cases as contact tracing using a test at a single timepoint with 80% power and a 95% confidence level, a total of 604 close contacts will be enrolled (302 per Arm). To achieve this, we anticipate needing to enroll approximately 150 index cases (75 per Arm), though enrollment of index cases will continue until desired number of close contacts has been enrolled.
**Number of participating sites**	1-2 sites (Porto Velho, Brazil and Curitiba, Brazil). The number of health units in the study will be based on the number of cases at the time of study initiation. Recruitment methods will be dynamic over the course of the study, to account for unpredictable changes in caseload.
**Study Duration**	Estimated 6 months
**Participant Duration**	Up to 10 days

1.0 Background and rationale for the study

The Coronavirus disease (COVID-19) pandemic, caused by SARS-CoV-2, has led to an unprecedented public health crisis. In Brazil, the first confirmed case of COVID-19 was detected on February 26, 2020 and the virus has since spread widely throughout the country, with over 25 million confirmed cases and over 600,000 deaths, placing significant burden on the country's health system.[1] SARS-CoV-2 infection can present in a broad spectrum of disease severity, ranging from asymptomatic, to mild symptomatic, to severe, to fatal. Among symptomatic patients, infection can trigger severe acute respiratory illness often characterized by flu-like symptoms (e.g., fever, cough, sore throat, headache, myalgia, fatigue).[2,3]

Insufficient testing continues to limit the effectiveness of the global response to the coronavirus disease pandemic. In many settings, the current standard for COVID-19 diagnosis is reverse transcription polymerase chain reaction (RT-PCR) detection of SARS-CoV-2 from nasopharyngeal (NP) swabs.[4] The limitations inherent in this testing method include availability of and access to reagents, swabs used for the collection of specimens, and the personal protective equipment needed to protect health care workers and patients when collecting NP and nasal specimens. Further, this molecular testing method is highly centralized. This centralization will continue to limit testing deployment options, making it harder to test oligosymptomatic and asymptomatic patients, close contacts of SARS-CoV-2-infected patients, and others who do not live in close proximity to central testing locations.

Rapid antigen tests for professional and self-test use offer multiple benefits in comparison to RT-PCR tests for the detection of SARS-CoV-2. They have been developed as both laboratory-based tests and for near-patient use (POC), with results generated in < 30 minutes and at low cost. They offer expanded access in places that do have molecular testing capacity and results can be returned quickly, facilitating faster results reporting and subsequent linkage to care. Most currently available rapid antigen tests show a lower sensitivity compared to RT-PCR, while their specificity is generally reported to be high. WHO recommends use of rapid antigen tests and self-tests and suggests that test kits should meet the minimum performance requirements of ≥80% sensitivity and ≥97% specificity in priority use cases/populations.[5–8] Further, rapid antigen

tests may be more suitable in settings where people have been previously infected and molecular testing methods may continue to return positive results due to residual viral fragments.

By expanding equitable access to quality, affordable diagnostics, health programs can better identify infections early, isolate cases of active infection, and provide effective case management.[9] Diagnostic strategies that are low cost, rapid, and easily accessible are critical to control the pandemic. While real-time polymerase chain reaction (RT-PCR) based assays remain the gold standard for the detection of emerging respiratory viruses, the need for high through-put virus detection has fueled development of Antigen-detecting rapid diagnostic tests (Ag-RDT), that can be performed at the point of care (POC).[10,11] Besides the performance of the test, other practical and strategic factors play significant roles in deciding if a test can be used and with which indications. Examples of these considerations are the timeliness of test results, the scalability, the simplicity of use, the human and material resources required and overall logistical arrangements for sampling and testing costs. The epidemiological situation will also affect the testing strategy, with certain methods being more or less suited to surges in cases and thus increased demand for testing.

Promising options that can serve to broaden testing include the use of self-tests.[12–14] Self-testing for COVID-19 may reduce the demand on health facilities while addressing many of the usual barriers to uptake of services, leading to timely testing of potentially infectious individuals. Earlier diagnosis can enable more timely isolation to minimize onward transmission and earlier clinical intervention, when needed, which can potentially improve individual patient prognosis, particularly given the availability of new antivirals that are more effective the earlier they are administered. Additionally, self-testing can reduce health care worker exposure to infectious material. Self-testing can be reliable and accurately performed in comparison to testing by trained professionals. It can achieve good sensitivity and specificity with minimal user errors. Self-testing as shown high levels of acceptability and can increase equity by providing more testing options. It can also be used to facilitate decision-making about participating in group activites in situations that may not warrant a clinical or laboratory test. In short, there is evidence that self-testing for COVID-19 is feasible and acceptable, with a WHO recommendation and with some specific products receiving emergency use authorization from the US Food and Drug Administration (FDA).[15] Based on the most up to date evidence on COVID-19 self-testing, the National Surveillance Health Agency (ANVISA) also recently authorized (RDC 595, 28 January 2022) the distribution and use of COVID-19 self-tests in Brazil.

While self-testing has the potential to contribute to the COVID-19 response, it also raises important concerns that need to be considered when this testing modality is implemented. Firstly, available antigen tests may have variable performance in asymptomatic individuals.[16,16] It is possible that performance may be further compromised when the antigen test is used for self-testing due to errors occurring through use by untrained users. False negative results may prompt infected individuals to stop self-isolation and thereby contribute to virus transmission, while false positive results may lead to unnecessary stress and anxiety and unnecessary absences from work, school and social activities. However, the recent expansion of self-testing for COVID-19 has demonstrated that the tests show good performance and can be used well.[15] Secondly, self-testing results may not be reported and be missed by local health authorities and the national surveillance system. Clear communication on actions for positive and negative results, relevant

support tools, efficient links to post-test counselling and easy access to results reporting are needed as key components of self-testing programs. The issue of results reporting is one of primary concern for key policy and regulatory stakeholders in Brazil. There are ongoing discussions regarding the best ways to facilitate results reporting and test case confirmation.

Contact tracing is the process of attempting to identify people who have recently been in contact with someone diagnosed with an infectious disease, especially in order to treat or quarantine them. Early on in the COVID-19 pandemic, contact tracing was identified as a potentially effective intervention for limiting the number of cases and linking at-risk individuals to testing and care.[17] Since then, both observational and modelling studies have shown that contact tracing is associated with better control of COVID-19. However, the impact of contract tracing is mediated by a number of factors, including the time it takes to identify and notify contacts and the number of positive cases that participate in contact tracing.[18] During period of peak transmission and many cases, local health systems responsible for contact tracing can become overburdened. Contact tracing efforts may be slowed, stymied, or abandoned all together when the number of cases exceeds the public health system's capacity to identify and follow-up with exposed cases. The responsibility of exposure notification may also be left to the positive case themselves. Tools, like access rapid testing, that lower the burden of contact tracing on the health system may help increase the effectiveness of contact tracing at the local level. In Brazil, contact tracing programs and the specifics of how to implement them are recommended in two national policies: 1) National Testing Plan and Epidemiological Surveillance Guide for COVID-19 and 2) the National Expansion Plan for Testing for Covid-19. These policies have been established by the national government and are implemented at the local or municipal level and the effectiveness of these programs has varied over the course of the pandemic, based on local context and epidemiology. [19,20]

In the context of contact tracing, rapid antigen tests can also allow for faster identification of infectious contacts.[21,21] Integrating rapid tests into public health services (especially primary healthcare) will be critical to mitigating the unequal effects of the pandemic and reaching at-risk populations. In Brazil, the primary health care system is widely used and works to ensure equitable access to health care. By integrating these tests into public health system programs and strategies rather than simply making them available as a consumer product, contact tracing with self-tests has the potential to screen at-risk populations, identify positive and potentially infectious individuals, and ensure key linkages to the necessary follow-up and treatment needed. Self-testing and serial self-testing may be advantageous to contact-tracing efforts, as it allows exposed individuals to monitor themselves over time and does not rely on a single time point to determine infection status.

As ANVISA authorized the registration of self-test kits in Brazil, manufacturers have been applying for market authorization and new products will be available in the market shortly.[2222] Currently, they are not part of any local national public health testing strategy. There is some uncertainly as to whether self-tests will be purchased and used within the public health system. The recent omicron wave, as well as anticipated waves, of the pandemic has only increased pressure to make more and diverse testing options available and easy to access. More evidence on the effectiveness and feasibility of self-testing is needed, particularly within specific use cases to understand how scarce resources can be best allocated to achieve impact. Self-testing as part

of a contact-tracing strategy presents an interesting opportunity to consider the impact of self-testing when integrated into a public health strategy and not as an alternative to public health utilization. The recommended on-label testing algorithm for many self-tests calls for testing twice in the event of a negative test. Much of the performance data supporting the use of antigen self-test is generated using this testing algorithm. However, exposed individuals may not seek care during the recommended time period post exposure (2-5 days) and that if they need to visit a health care provider for testing, they may not make a return visit for a second test if the first test indicates a negative result. Serial self-testing as a part of a public health system contact tracing strategy may be a viable strategy to avoid multiple follow-up visits on the part of health care providers or patients and benefit from the decentralized and flexible nature of self-testing.

To better understand the feasibility of self-testing as part of a contact-tracing system, we will conduct a mixed-methods pragmatic trial. The study has potential to inform policy and practice around COVID-19 self-testing in Brazil and globally, by informing optimal diagnostic algorithm and operational best practices.

2.0 Study objectives and endpoints

The primary objective of this study is to evaluate the effectiveness of contact tracing supported by serial self-testing among exposed individuals compared to contact tracing with a professional-use Ag-RDT performed at one visit.

Secondary objectives include the following:

- To evaluate the operational feasibility of self-testing within the public health contact tracing system
- To evaluate the concordance of supervised self-tests as compared to an Ag-RDT test conducted by a trained health worker
- To explore barriers and facilitators at the provider and patient levels that mediate use of COVID-19 self-testing and adherence to triage and treatment guidelines
- To generate supportive user resources for self-testing implementation

Table 1 summarizes study objectives and endpoints and lists the study/instrument used to capture them.

Table 1: Study Objectives and Endpoints

Objective	Endpoints	Study/Instrument
1. To evaluate the effectiveness of contact tracing supported by serial self-testing among exposed individuals compared to contact tracing with a professional-use Ag-RDT performed at one visit	1.1 Proportion of primary exposure close contacts who test positive per index case in Arm 2 compared to Arm 1	Main Study/Participant Follow-Up Survey
	1.2 Proportion of Ag-RDT positive close contacts per index case who report adhering to recommended treatment or isolation guidelines in Arm 2 compared to Arm 1	Main Study/Participant Follow-Up Survey

2. To evaluate the operational feasibility of self-testing within the public health contact tracing system	2.1 Proportion of exposed contacts in the self-testing group (Arm 2) who report test results as per local guidelines	Main Study Arm 2/Participant Follow-Up Survey
	2.2 Number of self-tests used by secondary exposures	Secondary Exposure Close Contacts Sub-Study/Anonymous Data Collection Form
	2.3 Proportion of exposed contacts who perform a self-test per manufacturer instructions, under supervision	Main Study Arm 2/Enrollment Visit
3. To evaluate the concordance of supervised self-tests as compared to an Ag-RDT conducted by a trained health worker	3.1 Concordance of self-test result with professional use Ag-RDT test result	Main Study Arm 2/Enrollment Visit
4. To explore barriers and facilitators at the provider and patient levels that mediate use of COVID-19 self-testing and adherence to triage and treatment guidelines	4.1 Barriers and facilitators to self-testing: acceptability, preferences, and user perspectives across stakeholders, including patients and health professionals	Focus Groups, Participatory usability workshop, Exit Interviews
5. To generate supportive user resources for self-testing implementation	5.1 Insights into user needs for instructions and training, Additional resources adapted with user input for self-test kits	Usability Workshop

Null hypothesis: Compared to routine COVID-19 contact tracing defined as screening with a professional use Ag-RDT at one visit, contact tracing with serial self-testing will not identify any additional COVID-19 positive cases over an approximately six-month period in Porto Velho, Brazil and Curitiba, Brazil.

Research hypothesis: Contact-tracing facilitated by serial self-testing identifies more positive COVID-19 cases among exposed close contacts than standard contact tracing over an approximately six-month period in Porto Velho, Brazil and Curitiba, Brazil.

3.0 Study design

3.1 Overall design

This is a two-arm, randomized pragmatic trial of a contact tracing program supported by serial self-testing with two additional sub-studies. Index cases will be randomized to Arm 1 (contact tracing by professional Ag-RDT at one timepoint for close contacts following exposure) or Arm 2 (serial self-test supported contact tracing for primary exposure close contacts). Serial self-testing in Arm 2 will *not* supersede any local, regional, or national policies regarding self-isolation or quarantine procedures. An overview of the study design is shown in Figure 1a.

Figure 1a: Main Study Design Overview

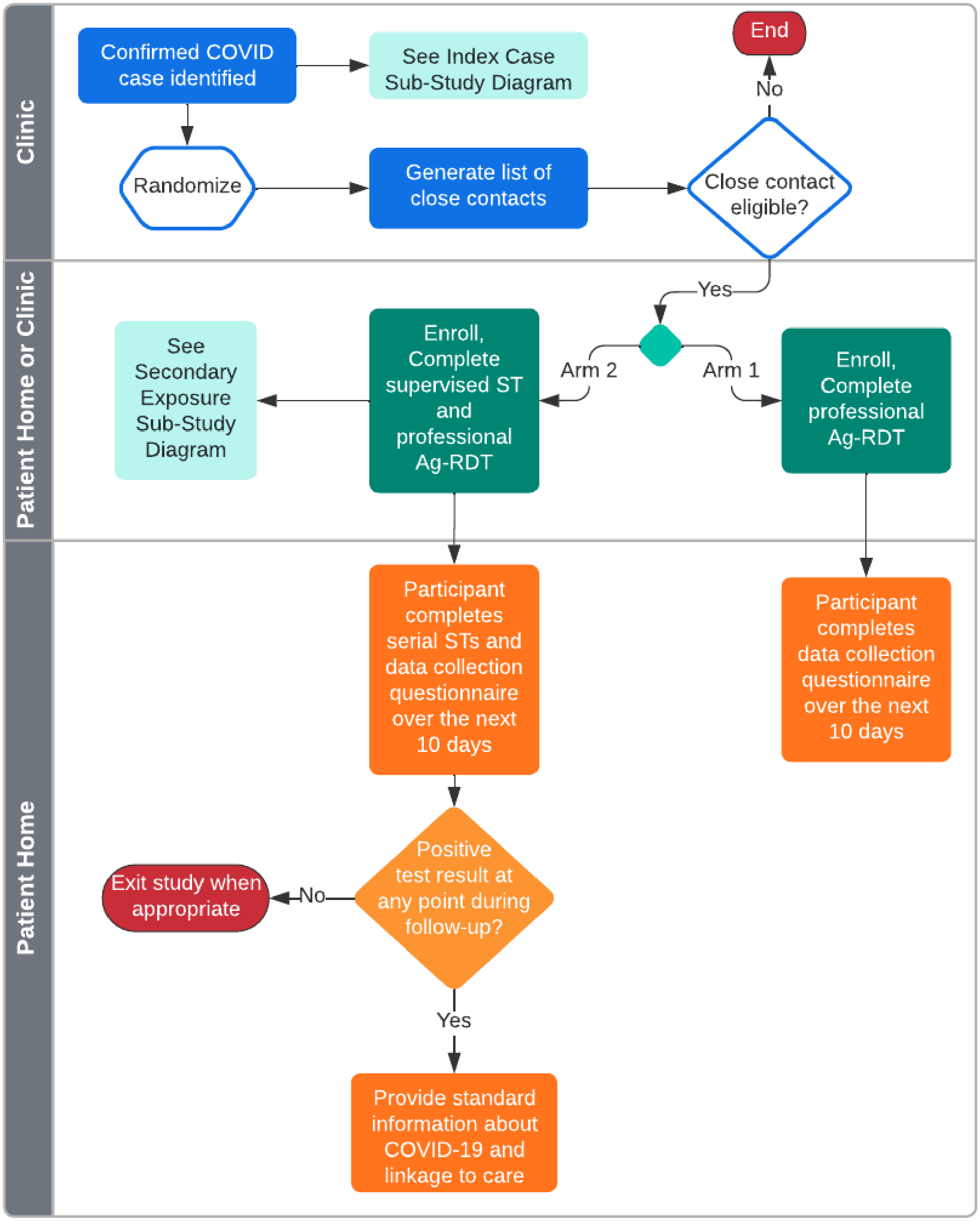

Health units and study staff will be trained on the use and supervision of self-tests. Index patients will be recruited through existing clinical care platforms in Porto Velho and Curitiba. These will include public health clinics, urgent care centers, and outpatient departments. Based on the

epidemiology and case load at the time of study start, additional recruitment platforms may be used, including any testing sites established to meet an increased demand for tests. Close contacts will be identified and recruited through a contact elicitation interview and contact tracing. All enrolled close contacts will be tested at enrollment by a health worker using an ANVISA approved antigen test intended for professional use. Close contacts in Arm 2 will also be supervised using an ANVISA approved COVID-19 self-test. Patient follow-up and linkage to care will be based on the results of the professional use antigen test. All participants in Arm 2 will continue through study procedures regardless of their test result in order to facilitate the secondary exposure sub-study and enrich the follow-up data collected.

Following consent, the index case will be asked by study staff to provide names and contact information of any close contacts they are comfortable sharing. The index case will also be randomized at this point, though their assigned group will only be known to study staff. Study staff will then reach out to each close contact and notify them that they have been exposed to COVID-19. Study staff will give the close contact information consistent with national policy at the time of study implementation regarding testing, masking, and isolation. Study staff will describe the study per randomized group to which the close contact belongs. If the close contact is interested in participating, study staff will confirm a time to enroll the close contact at either the close contact's home or the clinic. Study staff will consent the close contact at the enrolment appointment. If the close contact is in Arm 1, study staff will administer a professional Ag-RDT. The close contact will also complete a daily questionnaire for the duration of the 10-day follow-up period. If the close contact is in Arm 2, they will complete a self-test supervised by study staff and study staff will administer a professional Ag-RDT. The close contact will be given self-tests to use daily over the next 10 days. The close contact will be given additional self-tests to give to each of their household members. The close contact will complete a daily questionnaire in addition to the self-tests for the duration of the 10-day follow-up period.

An observational sub-study will be conducted among secondary exposures in Arm 2 (household members of primary exposure close contacts of the index case). Enrolled primary exposure close contacts will be given additional tests (up to 3 per household member) to provide to their household members along with guidance on how to use the tests. Anonymous data will be requested and submitted by users for any of these tests that are used. Figure 1b summarizes this sub-study. The primary goal of the observational sub-study is to understand how individuals at risk of infection who may have been exposed to SARS-CoV-2 opt to use or not to use self-tests. The tests will be provided free of charge with no prior instructions on testing frequency or cadence. These data will inform evidence gaps related to questions of operational feasibility of serial testing outside the context of a research study. Specific endpoints include the number of self-tests used by secondary exposures as well as the number of positive test results reported by secondary exposures.

Figure 1b: Secondary Exposure Close Contacts Study Design

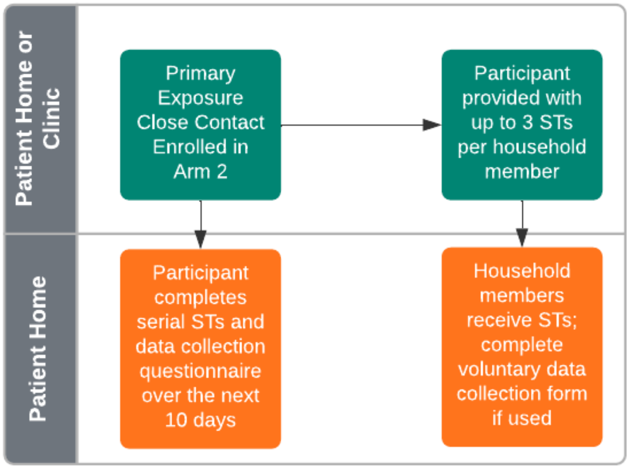

Index cases will also be followed to capture behavioral data about people who test positive for COVID-19 infection. Index cases will be asked to complete a daily questionnaire to provide information about their symptoms, isolation behaviors, and any other tests done. Figure 1c summarizes this sub-study.

Figure 1c: Index Case Sub-Study

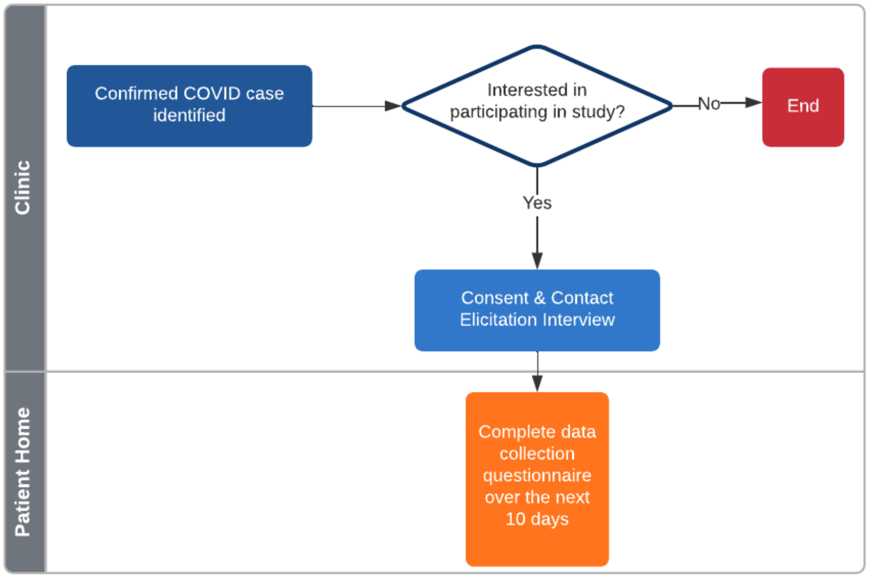

Planned data collection activities are outlined below in Figure 2.

Figure 2: Planned Data Collection Activities

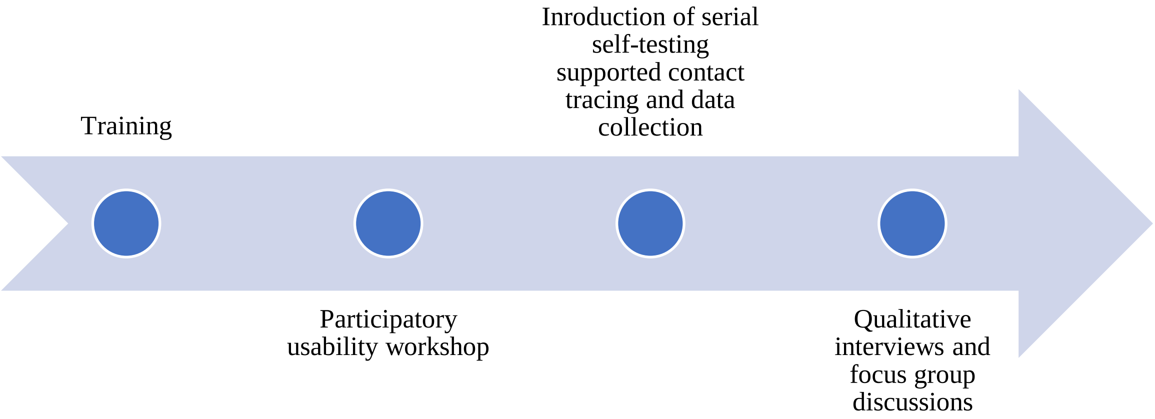

3.2 Qualitative and usability data collection

Secondary outcome measures will explore barriers and facilitators to contact tracing supported by self-testing. Barriers and facilitators will be explored using qualitative methods through focus group discussions with members of the health unit and study participants. A usability workshop will be conducted to develop appropriate supportive resources for self-testing with input from providers and potential end users.

3.32 Study Site

This study will take place in Porto Velho, Rondônia, Brazil, at the Centro de Pesquisa em Medicina Tropical de Rondônia (CEPEM) and a site in Curitiba, Paranà. Porto Velho is the capital city of Rondônia and the center of health care in the state with an estimated population of more than 529,000 people. Porto Velho has been significantly impacted by COVID-19. As of January 2022, there have been more than 90,000 confirmed cases of COVID-19 in the Porto Velho municipality, with approximately 80 to 400 new cases per day in the omicron wave.

CEPEM is an established clinical research center in Porto Velho that serves as a primary testing and treatment center as part of the SARS-CoV-2 response in Rondônia. The investigators at CEPEM have been working across multiple research streams to better understand SARS-CoV-2 diagnostic and treatment solutions and collaborate closely with Fiocruz Rondônia, a Brazilian governmental scientific institution focused on research and development in biological sciences and public health strategy with expertise in infectious diseases and established laboratory capacity in Porto Velho. CEPEM has also previously served as a clinical site for COVID studies and is responsible for technical supervision and training of teams working in primary care in the municipality. Therefore, this site has access to lower levels of health care service delivery.

The health system within Porto Velho is structured through health units that are responsible for providing care to a specific catchment area. There are 18 basic health units in urban areas and 31

in rural areas. Health units are generally staffed with nurses, technicians, community health workers, doctors, and pharmacists, with select additional specialty providers staffed based on the size and location of the health unit. All health units are equipped to handle basic patient needs, with additional abilities available depending on health unit size and location (e.g., management of severe COVID cases). Should a health unit need to provide advanced care that they are not equipped to handle, there is an Emergency Mobile Care Service that will stabilize the patient. Should the patient require hospitalization, they are transferred to the Rondônia Tropical Medicine Center. The city has also opened a few facilities to specifically serve patients suspected of COVID-19.

We will also be conducting study activities at a second site in Curitiba, Paraná, Brazil at Ouvidor Pardinho Health Unit (Unidade de Saúde Ouvidor Pardinho), located downtown Curitiba. This site provides a testing and treatment services through the public health system, currently conducting approximately 50 tests per day. Curitiba is the capital and largest city in Paraná located in southern Brazil with an estimated population close to 2 million people. Curitiba is also the home of various research institutes, including Fiocruz - Paraná, who have significant expertise in conducting research studies, and has been nationally and internationally recognized for having been the first city in the country to implement HIV self-testing in Brazil. In addition, conducting this study in Curitiba in addition to Porto Velho will increase the likelihood of reaching recruitment goals, especially given the unpredictable nature of COVID-19 prevalence.

4.0 Current practices and study interventions

The study aims to assess effectiveness and feasibility of a COVID-19 screening and diagnosis strategy. All decisions about clinical case management will be made through referral to the municipal health system

4.1 Diagnosis and case management standard of care

Professional use rapid antigen tests are widely used in Brazil. In September 2021, the Ministry of Health issued a the National Plan to Expand COVID-19 testing. The main objective of the policy is to expand timely access to COVID-19 testing using rapid antigen tests. In alignment with WHO recommendations, the policy recommends the use of these tests in three public health strategies:

- Diagnostics: to be used for symptomatic individuals/COVID-19 suspected cases
- Active search: to be used for contact tracing
- Triage process: to be used for asymptomatic individuals, especially those at higher risk.

Antigen tests have been procured and are widely available in the public health system being used according to the guidelines above, but actual implementation varies according to how testing is handled by State and Municipal health authorities. In April 2020, ANVISA issued a temporary and exceptional authorization for COVID-19 rapid tests to be performed in drugstores, which must be performed by a pharmacist. In January 2022, ANVISA approved the distribution and use of self-tests and is currently allowing the registration of self-tests under specific conditions.

RT-PCR remains the laboratory test of choice for the diagnosis of symptomatic patients in the acute phase (between the 3rd and 7th day of symptom onset) and may or may not be needed for confirmatory testing of Ag-RDTs.[23] Testing for COVID-19 in Porto Velho is usually done using RT-PCR on nasal, nasopharyngeal or oropharyngeal swab specimens, though antigen tests conducted by a health professional are also accepted. The turnaround time for RT-PCR test results is typically two to five days, which has placed limitations on the number of tests that are able to be run for suspected cases, particularly during times of high testing demand. As a result, RT-PCR testing is generally limited to patients with moderate or severe symptoms at the discretion of the care provider. Patients with mild symptoms may not receive confirmatory RT-PCR testing following a positive Ag-RDT result in Porto Velho and may instead be guided to isolate at home with remote monitoring.[24]

Clinical management of suspected and confirmed cases of COVID-19 in Brazil varies depending on the severity of the patient’s illness (mild, moderate, severe). Definitions of these classifications are provided in Table 2 below from Brazil’s national guidelines for the management of COVID-19 patients.

Table 2: National guidelines for classification of severity of COVID-19 signs and symptoms, by population group

<table>
<tr><th>Population</th><th>Mild</th><th>Moderate</th><th>Severe</th></tr>
<tr><td>Adults and pregnant women</td><td rowspan="2">Flu syndrome: cough, sore throat, or runny nose whether followed by:
- Anosmia (olfactory dysfunction)
- Ageusia (taste disorder)
- Runny nose
- Diarrhea
- Abdominal pain
- Fever
- Chills
- Myalgia
- Fatigue
- Headache</td><td rowspan="2">Persistent cough + daily persistent fever
OR
Persistent cough + progressive worsening of another symptom related to COVID-19 (adynamia, prostration, hyporexia, diarrhea)
OR
At least one of the above symptoms + presence of a risk factor</td><td>Severe acute respiratory syndrome, defined as flu-like syndrome with:
Dyspnea / respiratory discomfort
OR
Persistent pressure in the chest
OR
O2 saturation less than 95% in ambient air
OR
Bluish coloring of lips or face
* Important: in pregnant women, observe hypotension.</td></tr>
<tr><td>Children</td><td>-Tachypnea: ≥ 70 rpm for children under 1 year; ≥ 50 rpm
for children older than 1 year;
- Hypoxemia;
- Respiratory discomfort;
- Change in consciousness;
- Dehydration;
- Difficulty feeding;
- Myocardial injury;</td></tr>
</table>

			- Elevation of liver enzymes<br>- Coagulation dysfunction; rhabdomyolysis;<br>- Any other manifestation of damage to vital organs

For cases or suspected cases with mild symptoms, patients are typically advised to isolate at home and receive information from their health provider with guidance about signs and symptoms to monitor for up to 14 days after the onset of symptoms. Face-to-face assistance may be provided during the monitoring period, ideally at home. For moderate or severe cases, clinical management varies depending on the presentation of symptoms and may include, for example, hospitalization, oxygen therapy, mechanical ventilator support, and/or venous hydration.

4.2 Arm 1: Control Arm contact tracing

The control arm will utilize a common model of contact tracing: positive cases will be asked to provide a list of their close contacts they are comfortable disclosing, and those exposed individuals will be tested using a professional use Ag-RDT at one timepoint following exposure. This method of contract tracing is consistent with national policies, including the National Testing Plan and Epidemiological Surveillance Guide for COVID-19 and the National Expansion Plan for Testing for Covid-19 as well as the implementation of both policies through local municipal offices.

Standard contact tracing practices vary at the study sites based on health system capacity, current public health guidance, COVID epidemiology, and caseload. Throughout the pandemic, individuals testing positive for Covid-19 have been instructed to notify their close contacts and recommend they get tested 2-5 days post-exposure. During periods of low transmission and depending on health unit bandwidth, the public health system has taken on the responsibility of conducting the contact tracing by asking the confirmed positive index case for their contacts and contacting them on behalf of the participant. During period of high transmission, the number of individuals testing positive is generally too large and the public health system does not have the capacity to contact all exposed individuals. For the purposes of this study, this will be standardized in order to demonstrate the additive impact of self-testing. By standardizing the routine practice, the comparison between standard contact tracing and contact tracing with serial self-testing will be facilitated.

Primary exposure close contacts will be identified through a contact elicitation interview with the index case. Close contacts in Arm 1 will be tested using a professional use Ag-RDT at one time point and then asked to complete daily questionnaires to capture current symptoms and isolation and quarantine behaviors for up to 10 days following the date of enrollment. The daily questionnaires will also serve to provide information to the participants regarding local public health guidance for masking, vaccination, isolation and quarantine.

4.3 Arm 2: Study intervention

In the interventional arm, the study will use serial self-testing to bolster contact tracing efforts. This arm will use Ag-RDT self-tests currently registered for use in Brazil by ANVISA and Ag-RDT that are currently registered for professional use.

Self-tests will be used first under supervision and followed by un-supervised self-testing on subsequent days. The supervised self-test will serve as a quality check to ensure the validity of subsequent unsupervised test results. All test operators, including study staff and participants, will be provided training or guidance documents to ensure appropriate use. All participants/ test operators will be observed prior to using the test independently. Participants/ test operators may receive additional guidance in appropriate use of the test as necessary. Any participants/ test operators who are unable to appropriately conduct the self-test after a reasonable training effort (i.e., unable to obtain a result after three attempts with staff assistance) will be withdrawn from the study and referred for standard follow-up.

Primary exposure close contacts will be identified through a contact elicitation interview with the index case. Close contacts in Arm 2 will be tested using a professional use Ag-RDT and asked to conduct a supervised ST at their home or at the clinic. The order in which samples are collected will alternate based on participant ID to account for sample depletion. Close contacts will then be given up to 10 STs to complete daily for up to 10 days following the date of enrollment. Participants will also complete questionnaires to capture their ST results, current symptoms, isolation behaviors, whether they distributed additional STs to their household members or live with an index case, and usability data about the STs.

4.3.1 Index case interventions

Participants testing positive for COVID-19 by any approved test will be recruited into the study. Potential participants may be screened using a rapid test and their test result shared with study staff for the purposes of recruitment. Consent will be sought if this screening is done by study staff rather than as part of routine clinical care. Index cases will be asked to participate in a contact elicitation interview where they provide information about their close contacts prior to symptom onset or positive test result. Index cases will also be asked to complete daily surveys to capture current symptoms and isolation behaviors for up to 10 days following the date of enrollment.

4.3.4. Secondary exposure intervention

Household members of primary exposure close contacts in Arm 2 will also have the option to participate in an anonymous observational study. Primary exposure close contacts in Arm 2 will be provided with several STs to give to their household members for use as they see fit. We will request users to submit anonymous data for each ST used, including the test result, motivation for use, and any symptoms.

During the enrolment visit, primary contacts enrolled in Arm 2 will be asked how many people are in their household. They will be given three times that number to take home and instructed to give three tests to each member of their household. Each test will have a QR code and link to complete the survey with a caption requesting that the individual who uses the test submit their results and complete a short survey about their use of the test. The link will be to a public survey hosted through REDCap and no identifiable information will be collected.

Table 4: Summary of interventions by participant group

Intervention	Participant Group

Contact elicitation interview and request for follow-up data	Index Cases
Professional use Ag-RDT at enrollment and request for follow-up data	Primary Exposure Close Contact – Routine Care (Arm 1)
Professional use Ag-RDT and supervised ST + Serial ST (up to 10 days after enrollment)	Primary Exposure Close Contact – Intervention (Arm 2)
Ad-hoc and anonymous use of STs	Secondary Exposures (Arm 2 Only)

4.4 Tests used in the research activities
All of the rapid tests used in the study will be registered and approved for use by ANVISA. Specific lots of the self-tests distributed for use as part of the study intervention will undergo all relevant quality assurance procedures as recommended by ANVISA, the manufacturer, and the municipal health authorities.

5.0 Research participants
<u>Characteristics of research participants</u>
Participants for this study will include index cases with confirmed COVID-19 and their close contacts. Eligible index cases will be recruited through existing clinical care platforms, including platforms normally used by the health system such as the electronic Sistema Único de Saúde in Porto Velho and Curitiba. Close contacts will be identified through a contact elicitation interview that will be administered to the index cases at enrollment.

Index cases and close contacts will include adults and children 7 years of age and older.

5.1 Index Cases
<u>Inclusion Criteria</u>
- 7 years of age or older
- Positive screening result for SARS-CoV-2 infection according to an ANVISA-approved Ag-RDT or RT-PCR
- Willing and able to provide informed consent (or assent with parent/legal guardian consent if <18) to participate and comply with study requirements

<u>Exclusion Criteria</u>
- Any study site employees who are involved in the protocol or may have access to study-related data
- Treating clinician deems inappropriate to enroll
- Previous study participant

5.2 Primary Exposures
<u>Inclusion Criteria</u>
- 7 years of age or older and meets age indication per the self-test’s instructions for use
- Exposure (within 6ft/1 meter for more than 15 minutes, physical contact, or healthcare worker caring for patient without appropriate personal protective equipment) to an

enrolled index case in the 2 days prior to symptom onset of the confirmed index case or within 7 days of the index case's positive COVID-19 test
- Willing and able to provide informed consent (or assent with parent/legal guardian consent if <18) and comply with study requirements

Exclusion Criteria
- Contraindication to nasal swab
- Any study site employees who are involved in the protocol or may have access to study-related data
- Previous study participant

5.3 Usability workshop participants

Inclusion Criteria
- 18 years of age or older
- Considered to be an intended user of COVID self-tests
- Willing and able to provide informed consent

Exclusion Criteria
- Study site employee involved with the protocol

5.4 Focus group participants

Inclusion Criteria
- 18 years of age or older
- Considered to be a relevant stakeholder in future practices or policies around the implementation of STs (e.g., patient, local health department official, implementer of COVID ST strategy for public health)
- Willing and able to provide informed consent

Exclusion Criteria
- Study site employee involved with the protocol

6.0 Study procedures

Please see Figure 3 for a diagram of study procedures by arm. All participants will be tested using a professional Ag-RDT at the enrollment visit; participants in Arm 2 will also complete a supervised self-test. Participants in Arm 1 will be provided standard follow-up and participants in Arm 2 will conduct a self-test each day for up to 10 days post-enrollment. All participants will be asked to complete daily remote assessments of symptoms as well as any test results and health behaviors. Participants may opt to submit assessments online or by calling the study team. If a participant does not complete the assessment for two consecutive days, a study team member will contact them to complete the assessment(s).

Figure 3: Study Procedures by Participant Group

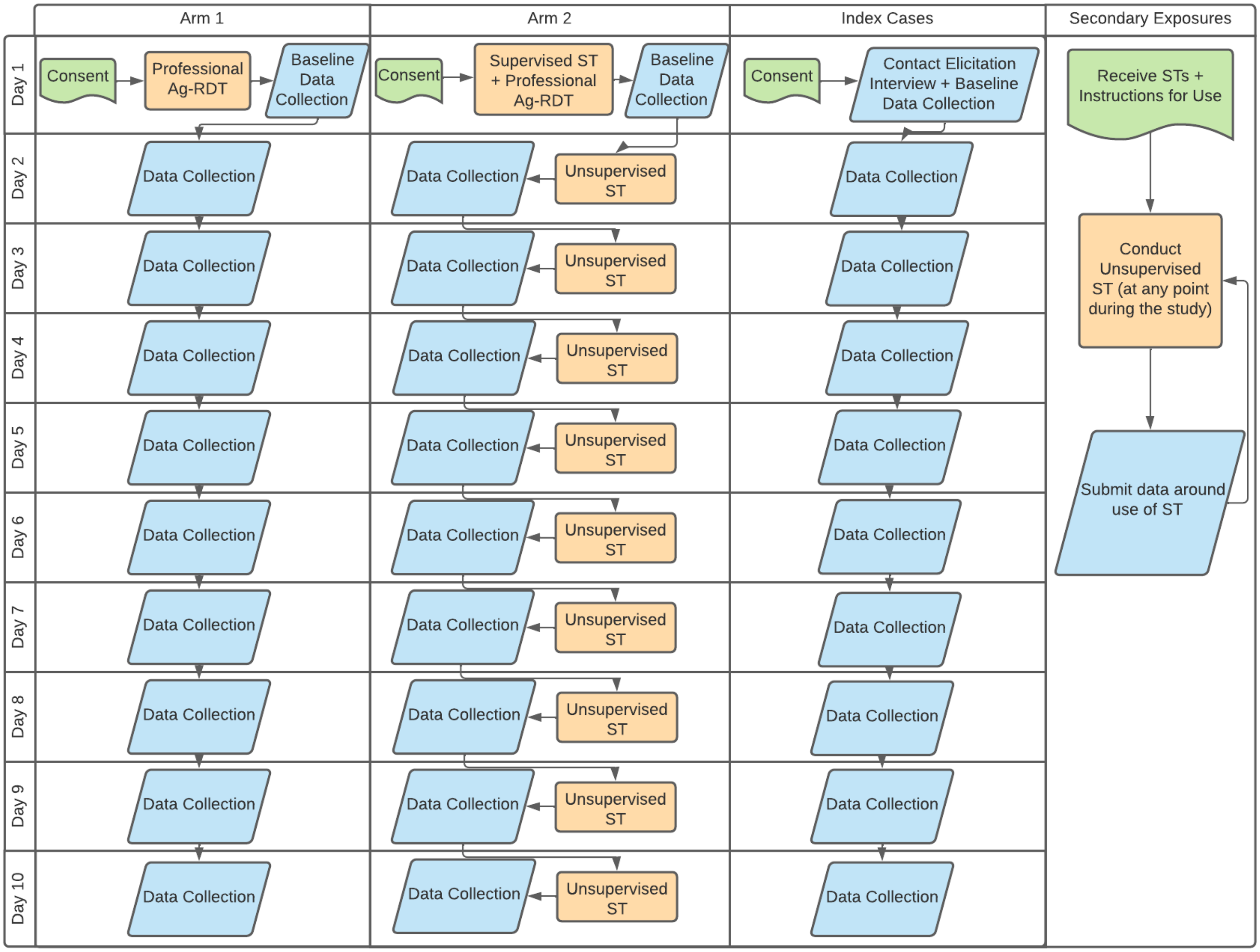

Data collected at the baseline visit will include demographic information, health history, current symptoms, and Ag-RDT test result(s). This information will be used to describe the patient population and in the primary analysis. Data collected through follow-up surveys will include current symptoms, isolation behaviors, use of other COVID-19 tests, and ST results (if applicable). This information will be used to describe the population and in the primary analysis. Information collected through index case follow-up will include current symptoms and isolation behaviors; this data will be used in the index case sub-study. Information collected through anonymous survey completion by secondary exposures will include use of self-test, result of self-test, reason for use of self-test, current symptoms, and isolation behaviors; this data will be used in the secondary exposure sub-study.

The survey will also include patient information at it relates to national and local guidelines for COVID-19 care and treatment, including any recommendations for isolation and quarantine.

This information will be updated to reflect current guidelines at the start of recruitment to ensure the safety and quality care of study participants.

6.1 Index Cases

6.1.1 Recruitment

Index cases will be recruited through existing clinical care platforms in Porto Velho and Curitiba. An Institutional Statement signed by the Municipal Department of Health from each site will be presented for the participant's recruitment. Patients from local healthcare facilities and outpatient clinics who are presenting with signs and symptoms consistent with COVID-19 will be pre-screened against the study eligibility criteria. Presenting patients to local healthcare facilities who have tested positive for COVID-19 will also be pre-screened against the study eligibility criteria. Individuals who are 7 years of age or older and have a positive COVID-19 test will be invited to participate in the study. Recruitment will be conducted in such a way as to not interfere with the potential participant's care.

6.1.2 Consent

Following recruitment, study staff will screen participants for eligibility either at the facility or through a home visit by study staff. The study team will explain the study and invite the person to participate. If the person expresses interest in participating in the study, she or he will be directed to a private space for consenting. Written consent will be obtained from all participants. See detailed consent procedures and explanation of ethical considerations in sections 7.0 and 12.0. This protocol will follow the CEP/CONEP System Resolutions in Brazil.

6.1.3 Procedures at the point of care

Following consent, study staff will collect basic demographic data (e.g., sex, age, ethnicity) and relevant information on health status/medical history from participants. A case investigation questionnaire will also be administered, which will include a contact elicitation interview. Index cases will be asked to complete a daily questionnaire for the following 10 days to capture information about symptoms and isolation behaviors.

6.2 Primary Exposures/Close contacts

6.2.1 Recruitment

Following identification of SARS-CoV-2 infection in an index case, all associated close contacts identified during the subject's contact elicitation interview will be notified of their exposure by phone within 72-hours. The identity of the index case may or may not be disclosed to their close contacts, depending on the preference of the index case. For each index case, associated close contacts will be screened against the eligibility criteria and recruited for participation in the study. De-identified demographic information will be collected and kept for all close contacts who are contacted, including age (<89), race/ethnicity, and enrollment status (including reason for refusal if they decline to participate in the study, if available). All close contacts, regardless of their participation in the study, will receive standard guidance in accordance with national and municipal guidelines. For contact tracing, that will include information about their potential exposure, instruction on recommended isolation procedures, information on when/how they

should seek care, and information on resources available for their care and testing from the municipality.

During the notification/recruitment phone call, study staff will ask them to visit a clinic or schedule a time for a home visit.

All information collected during the contact elicitation interview will be considered protected health information and secured accordingly. Close contacts will be elicited through a contact elicitation interview and documented in log. Close contacts who agree to participate will be enrolled and their identifiable info stored in the enrollment log. Close contacts may refuse to enroll in the study. All close contacts reached by the study team will be given standard information regarding COVID-19 exposure in accordance with national and municipal guidelines. Their identifiable information will be destroyed if they decline to participate in the study. This method of screening via contact elicitation interview has been done previously at the site and has been found to be acceptable by national and local regulatory authorities (i.e., CONEP).[25]

Close contacts will be elicited from minors as part of a public health contact tracing program, in accordance with local and national Brazilian guidelines. All minors will only report contacts after the research team has obtained their assent and their parents' consent. The contact elicitation interview will occur in the presence of their parent/legal guardian to minimize feelings of coercion. They will be instructed to share any close contacts they are comfortable sharing. Study staff will make clear to minors that reporting their contacts is voluntary and will not interfere with their participation in the study or their clinical care.

6.2.2 Screening and consent

At the clinic or home visit, potential participants will be screened again against the eligibility criteria. The study team will explain the study and invite the person to participate. If the person expresses interest in participating in the study, she or he will be directed to a private space for consenting. Written consent or assent will be obtained from all participants. See detailed consent procedures and explanation of ethical considerations. This protocol will follow the CEP/CONEP System Resolutions in Brazil.

6.2.3 Procedures at the point of care

Study staff will collect basic demographic data (e.g., sex, age, ethnicity) and relevant information on health status/medical history from participants. A professional Ag-RDT will be done on each participant. Additionally, participants in Arm 2 will be asked to conduct a supervised self-test under observation. See detailed discussion of supervised self-test in section 4.2 Interventions.

6.2.4 Follow-up procedures

Enrolled close contacts will be enrolled into Arm 1 (routine contact tracing) or Arm 2 (serial self-testing) based on the Arm to which their associated index case was randomized. In Arm 1, participants will be asked to complete a health questionnaire remotely every day until day 10 that will include questions related to the onset of any relevant symptoms and isolation behaviors. In Arm 2, participants will be asked to complete a health questionnaire remotely every day until day 10 as well as conduct a self-test. All participants with positive self-test results will be referred for

appropriate care according to the standard of care and local public health guidelines in Porto Velho and Curitiba. They will also be asked to report their test results according to the local policies established at the time of study start. Participants in this arm will be asked to continue testing over the 10-day period with the self-test irrespective of if and when the test converts to positive. Their reported symptoms will be monitored and if the reported symptoms indicate a possible progression to moderate or severe illness, they will be referred to a health facility for immediate care. On the last day of follow-up, participants will be invited to share their GPS location data from their mobile phone. Consent will be obtained to share this data, which is optional and completely voluntary. All measures will be taken to guarantee participants' confidentiality and privacy.

6.3 Test result return and follow-up

All participants will receive their result from the ANVISA-approved Ag-RDT at the point of care and will be counseled based on their results. Any participant who tests positive for SARS-CoV-2 infection at any point will be referred for care according to local public health guidelines. All clinical management of patients based on the results of study tests will take place through the public health system.

All close contacts will be advised to follow local public health guidelines and isolation procedures.

All results of diagnostic tests for COVID-19 conducted as part of the study will be reported by CEPEM and ICC to Brazil’s surveillance notification system as required by local and national guidelines.

6.4 Secondary Exposures

Secondary exposures (household members of primary exposure close contacts enrolled in Arm 2) will be provided with several self-tests to use as they see fit. We will request a data form be submitted for each self-test that is used; this data will be anonymous. Participants will be made aware that use of the STs is completely voluntary, as is submitting anonymous data. Each ST kit will come with accompanying guidance on the optimal time to perform the ST, how to perform the ST, where to report a positive result, and what to do for a positive or negative result.

6.5 Usability

In addition to the main study, index cases, primary exposure close contacts, caregivers of primary exposure close contacts, and health workers will be given the option to participate in the usability study, provided they meet the eligibility criteria outlined in this protocol. Participants interested in the usability study will be consented in a private setting and enrolled into the study. Usability workshops will happen towards the beginning of the main study and in parallel so the materials can be refined and ready to hand over to appropriate stakeholders. Usability workshops will be conducted in Portuguese and all responses will be translated and reviewed in English.

Each participant will be provided with standard training materials about the appropriate use of the self-test. These materials will be developed in collaboration with end users. Following review of these materials, participants will be asked to perform a self-test. Study staff will observe the participant and conduct a task analysis. Following use of the self-test, the participant will be

given a questionnaire to assess test usability. Study staff will emphasize to participants that the questionnaire is being used to assess the effectiveness of the training content and test instructions and not the skills or performance of the user. Multiple types of self-tests may be evaluated depending on what is currently approved in Brazil at the time this study is running.

The usability sessions will be audio/video recorded to allow for optimal notetaking and ensure all relevant information is captured appropriately.

6.6 Focus Groups

Study staff will identify up to fifteen individuals based on their role in the public health system and experience with the study intervention. Local, regional, and/or national officials managing the COVID-19 situation will be purposively recruited based on study team/investigator relationships. Health workers will be recruited from local urban and/or rural health units. Study staff will contact selected individuals, explain the study, and invite them to participate in a private setting. If the individual expresses interest, they will be consented and enrolled into the study. Focus groups will happen towards the end of the main study and may continue beyond the final recruitment of participants into the main study. The focus groups will focus on implementation barriers and facilitators of the intervention.

Focus group discussions will be conducted with small groups of participants, facilitated by a trained study staff member, and last approximately one hour. Groups will contain no more than five participants, and up to three sessions will be held to reduce the risk of potential COVID-19 transmission among participants and staff. Focus group discussions will be conducted in Portuguese and transcripts will be translated and analyzed in English. Participants will be asked to share their opinions on potential use cases for self-testing in COVID-19, including barriers and facilitators to those use cases, with a particular focus on serial self-testing and the tested intervention. As available, protocols, policies, and other relevant supporting documentation may also be reviewed and analyzed.

The focus group discussions will be audio recorded to allow for optimal notetaking and ensure all relevant information is captured appropriately.

7.0 Consent process

7.1 Index Cases and Primary Exposures

Written informed consent or assent will be obtained from all index cases and primary exposures. Consent will occur in a private place in the home or at the healthcare facility. Members of the study team trained in the protection of human subjects will conduct the informed consent discussion. Study staff will review the study details with the potential participant. The potential participant will be given an opportunity to review the informed consent form and ask questions. Consent will take place in Portuguese; the informed consent form will be prepared in English and translated into Portuguese. When appropriate, a conversational style oral presentation of consent information will be made in the local language to participants to account for any difficulties understanding written consent forms due to low literacy. Parent permission will be obtained for all participants under the age of 18. Child assent will be obtained for all participants

between the ages of 7 and 17. Children aged 7-17 will express their assent in writing in the presence of their parent as a witness, to ensure the assent process is without any coercion.

During the consent process, the study team will explain the purpose of the study and what participation will entail. It will be emphasized that participation is voluntary and that their decision will not negatively affect the care they receive in any way. The informed consent form will review the study purpose, the procedures involved in participation, the potential participant's rights to withdraw, confidentiality, and benefits and risks of participating in the study. The potential participant will have the opportunity to ask questions. Consent for participation will be documented on a written informed consent form. One copy of the signed informed consent form will be provided to the participant and one copy will be kept for study records.

If a potential participant is illiterate, an independent, impartial literate witness will be asked to join the consent process. This witness will be a health care worker or other family member/neighbor uninvolved in the study. The study staff will read the consent form aloud to the potential participant and the witness will verify that the information read aloud matches the information written on the consent form. The witness will affirm that the study participant chose to be in the research study, that he or she was present the whole time the study was being explained, and that the participant had a chance to ask questions. The participant will get a copy of this form to keep. The witness will also sign the consent form.

Primary exposures will consent to participate in the study after the study team contacts them via phone. The purpose of this initial contact via phone is to 1) notify them of their exposure and 2) introduce the study. The study team will obtain their name and contact information through a contact elicitation interview with the index case, as per standard contact tracing protocols. This information will be considered protected health information and secured accordingly. If the primary exposure does not wish to participate in the study after learning about it on the phone, their name and contact information will be removed from all study documents. It is not practical to consent primary exposures to obtain and use their name and contact information for screening purposes, and they would need to be notified of their exposure anyways.

7.2 Secondary Exposures

Informed consent will not be obtained from secondary exposures, as the information being collected is anonymous and the research team will not have prescribed contact with these potential participants. It will be made clear that use of any self-tests and submitting any data is completely voluntary.

7.3 Usability Workshop and Focus Groups

Written informed consent will be obtained from all participants taking part in the usability workshop and focus groups. Consent will occur in a private place. Members of the study team trained in the protection of human subjects will conduct the informed consent discussion. Study staff will review the study details with the potential participant. The potential participant will be given an opportunity to review the informed consent form and ask questions. Consent will take place in Portuguese; the informed consent form will be prepared in English and translated into Portuguese. When appropriate, a conversational style oral presentation of consent information

will be made in the local language to participants to account for any difficulties understanding written consent forms due to low literacy.

7.4 Consent to Share GPS Location Data

Participants who would like to share their GPS location data from their phone will complete an additional consent form on the last day of follow-up. All measures will be taken in order to guarantee participants' confidentiality and privacy.

This consent form is already in use by the parent project for this activity, led by Dr. Helder Nakaya, Professor of Pharmaceutical Sciences at the University of São Paulo. The parent project for this activity is entitled "Evaluation of the Transmission Dynamics of the New Corona Virus (SARS-CoV-2) Using GPS Data" and has been approved by the CEP/CONEP System.

8.0 Statistical Considerations

8.1 Overview and General Design

This is a randomized trial to evaluate the effectiveness of serial self-testing to support contact tracing. Index cases will be randomized 1:1 to Arm 1 or Arm 2 to determine which study procedures their close contacts will follow.

8.2 Sample Size: Effectiveness

This study is powered to demonstrate a 7.5% difference in positive cases identified between Arm 1 and Arm 2. Prior work at this site with COVID testing of close contacts yielded a 30% PCR positivity rate among close contacts during a period of low to moderate transmission. To account for increases in vaccination coverage, high rates of prior infection, and the likelihood of low transmission following the Omicron wave, we estimate this population will have a 20% test positivity rate. Based on the established performance characteristics of Ag-RDT tests, we estimate serial self-testing will identify up to 75% of those cases (p_1 = 0.15 ∴ p_0 = 0.075).

Utilizing Equation 1 listed below, where $z_{\alpha/2}$ = 1.96 and z_β = 0.842, we calculate a total of 550 participants needed to complete the study (275 per Arm). To account for attrition in longitudinal data and the exclusion of unevaluable cases, we will increase the sample size estimate by 10% to enroll a total of 604 close contacts (302 per Arm). To achieve this, we anticipate needing to enroll approximately 150 index cases, with each index case yielding an average of 4 close contacts (75 per Arm), though enrollment of index cases will continue until the desired number of close contacts is enrolled. The average number of close contacts per index case is based on prior work at this site.[25]

Equation 1: Sample Size Calculation for a Difference in Proportions

$$N = \frac{2\left(z_{\alpha/2} + z_\beta\right)^2 * (p_0(1 - p_0) + \ p_1(1 - p_1))}{\Delta^2}$$

The primary analysis dataset will contain primary exposure close contacts in both Arms, with the exclusion of any primary close contacts who have recently tested positive for COVID-19 prior to

enrollment in the study. Secondary analysis will be conducted on index cases and secondary exposure close contacts from each sub-study.

8.3 Analytical methods: Effectiveness and operational feasibility

Participant characteristics will be compared between Arms and sites. Endpoints will be summarized using descriptive statistics overall, by Arm, and by site. Where appropriate, endpoints will be compared between Arms using T-tests or Chi-squared tests. Subjects with missing outcome data will be excluded from analysis; missing data will not be imputed. For the purposes of analysis, participants in Arm 2 will be considered to have complete primary endpoint data if at least 3 ST results are submitted over the 10-day follow-up period; for participants in Arm 1, having a professional Ag-RDT test result available at the enrollment visit will be considered complete. A binomial logistic regression will be conducted to evaluate the difference in close contacts per index case testing positive for SARS-CoV-2 infection between Arms. Analysis will be conducted on a modified Intention-to-Test (mITT) population with a Per Protocol (PP) analysis run for sensitivity. The mITT population will exclude close contacts who recently tested positive for COVID-19. We will reject the null hypothesis of no difference between groups if $p<0.05$.

Descriptive statistics and proportions will be used to describe the operational feasibility endpoints.

8.4 Concordance of Self-Tests with Professionally Administered Ag-RDTs

For the purposes of this analysis, an individual will be considered positive for SARS-CoV-2 infection if they test positive by a professional use Ag-RDT. The concordance of the supervised Ag-RDT will be determined by comparing the result of the supervised self-test with the result of the professional test. Test results will be classified as either positive or negative and percent agreement will be calculated (number of agreements/total number of test pairs x100).

To calculate operational performance, device failure percentages will be calculated as the total number of invalid tests divided by the total number of tests performed and then multiplied by 100%.

8.5 Sample Size and Analytical Methodology: Usability Study

Sample size for the Usability Workshop will be 6-10 participants.[26] The usability questionnaires will include both multiple choice and open-ended questions. Survey questions will be used as a framework for the analysis. Descriptive statistics and thematic coding will be used to summarize results. Qualitative data will be assessed using thematic analysis. If saturation of findings is not achieved, additional participants may be identified, up to the maximum enrollment stated.

8.6 Qualitative analysis of intervention barriers and facilitators

Sample size for focus groups will be up to 15 participants. Focus group discussions will take place in Portuguese. Data from focus groups will be transcribed and analyzed using a qualitative data analysis software. Transcripts will be translated into English and reviewed by study staff to identify key themes using inductive coding. A subset of constructions from the Consolidated Framework of Implementation Research (CFIR) will be adapted and used to develop the qualitative interview and focus group discussion guides.[27] The CFIR framework will then be

used to guide data analysis. If saturation of findings is not achieved, additional participants may be identified, up to the maximum enrollment stated. Demographic information will be collected to ensure that a wide variety of participants is included.

8.7 Documentation of commodity costs for self-testing supported contact tracing
This study will document the commodity costs required to implement a self-testing supported contact tracing program. Given the costs associated with such frequent testing, this intervention is unlikely to be adopted without adaptation. The commodity costs will be useful to inform any future adaptations of the intervention that may be implemented and as such will be tracked over the course of the project.

9.0 Confidentiality and data management

The investigators will ensure that all participants' confidentiality is maintained. Participants will not be identified in any publicly released reports of this study. All records will be kept confidential by each site. PATH will not have access to any records that directly identify the research participants. Each site will maintain a secure linking log that links participant study identification numbers to their name and contact information. The sample is expected to be large enough that combinations of demographic information will not identify participants. Additionally, there are no known rare ethnicities in Porto Velho or Curitiba that would increase the risk of certain participants being identified.

9.1 Data collection, entry, and quality control
Data will be entered by study staff and participants directly into an electronic, password-protected study database. Caregivers will enter data on behalf of children enrolled into the study. Electronic study records will be de-identified upon completion of data collection. The electronic records will be maintained indefinitely in the databases and remain password protected. PATH will not have access to any identifying information from participants, and the participant's name and contact information will not be included in the electronic study database. The electronic data collection system will have built-in quality checks and data will be reviewed regularly for quality and to prompt participate follow-up as needed.

Data will be collected from participants during the enrolment visit and then daily for the 10-day follow-up period. During the enrolment visit and following consent, study staff will ask the participant questions and record their responses on a data collection form. This may be a paper form that gets entered into an electronic database later or data may be entered directly into the electronic database. During the 10-day follow-up period, participants will complete a daily survey that gets automatically sent to them via email or text. They will complete this survey themselves online unless they opt for a member of the study team to call them and complete the questionnaire, in which case the study staff will enter their responses directly into the electronic database.

9.2 Data Access and Storage
The participants will be identified by a study identification number. The log linking the participant's name and contact information to the study identification number will be maintained

by the study staff at each site, and PATH will not have access to the link. All records will be kept locked and only accessible by a small team of study staff who must access the information (e.g., to complete participant follow-up); all databases will be password protected such that clinic staff and study staff will have access to their respective databases. Access to study data will be granted to authorized representatives from the sponsor, investigator institutions, and ethical or regulatory authorities to permit study-related monitoring, audits, and inspections as needed. These access terms are described in the consent forms.

Each site will maintain, and store securely, complete, accurate and current study records throughout the study. In accordance with regulations, study staff will retain all study records on site for at least three years after study closure. Study records will not be destroyed prior to receiving approval for record destruction from the sponsor. Applicable records include source documents, site registration documents and reports, informed consent forms, and notations of all contacts with participants.

All efforts will be made to minimize the amount of personally identifiable information recorded during the usability sessions and focus group discussions. Only restricted staff will have access to the data. All identifiers will be removed from the recordings when transcribed.

9.3 GPS Data

Participants who consent to share their GPS data will be given instructions to follow to upload their location data to a secure server. Only geographic locations, dates, and times will be submitted to the server; no personal information will be collected. The GPS data will be associated with a random number that is linked to the participant's study ID.

10.0 Study and safety monitoring

We anticipate that this study poses minimal risk to participants, as it does not involve any medical intervention and biological sampling is within acceptable ranges. No data safety monitoring board will be used. PATH, CEPEM, and the Curitiba site will conduct necessary staff training on any study procedures prior to initiating the study. Only research staff who have been trained in best practices for specimen collection and infection prevention will be involved in specimen collection.

The study team will be supervised by the local study lead. Study data will be aggregated into a database and a monitoring report will be generated regularly summarizing key indicators for study compliance. PATH, CEPEM, and the Curitiba site will hold regular study review calls to discuss data collection and data quality to date. These indicators may include but are not limited to the number of participants consented, the number of samples acquired, any deviations from study procedures, and corrective actions taken.

PATH will designate trained and qualified personnel to monitor the progress of this clinical study in accordance with study-specific SOPs. Prior to study start, a study training will be conducted to train staff on the protocol, the completion of study documentation and data collection forms, the monitoring schedule, and all regulatory requirements. A PATH

representative may conduct remote (or in-person, if feasible) site monitoring visits as needed to ensure compliance with the protocol and relevant SOPs.

The study will be conducted in accordance with the current approved protocol, International Conference on Harmonization (ICH) good clinical practices (GCP), relevant regulations and standard operating procedures. Regular monitoring will be performed according to ICH GCP. Data will be evaluated for compliance with the protocol and accuracy in relation to source documents.

10.1 Care for injury

In the unlikely event of a research-related injury, cost of treatment will be covered by the study, without the participant having to assume any expense.

11.0 Ethical Considerations

11.1 Risks and mitigation of risks

Study procedures do not represent significant risks to the participants beyond those that are associated with a nasal swab, such as pain, discomfort, and nosebleed. The risks associated with nasal swabs will be mitigated through user training on proper sample collection using a nasal swab. All decisions regarding clinical care will be made through referral to the local public health system. The study will review data in real-time to ensure that any information provided on clinical symptoms is referred appropriately per local public health guidelines.

There is a risk to study staff of COVID-19 exposure when performing the RDTs and interacting with primary exposure close contacts during the enrollment visit. There may also be a risk to close contacts of going to the clinic if they would not have normally done so to get tested or from allowing a healthcare provider/study staff member into their home. All study team members will adhere to strict and standard institutional procedures for infection control and will be provided with adequate personal protective equipment throughout the duration of the study. Additionally, any staff members with symptoms of COVID-19 or exposure (within 6ft/1 meter without wearing appropriate personal protective equipment) to someone with confirmed COVID-19 shall not interact with participants until deemed non-infectious by an appropriate clinical authority to mitigate the risk of transmission.

There is a minimal risk that participants recruited for the usability study and focus groups may feel compelled to participate in the study. We will mitigate this risk by communicating that participation is voluntary and interview questions will not cover sensitive or personal information.

All efforts will be made to maintain confidentiality and data security to mitigate the risk of breach of confidentiality. Study staff will participate in training for study confidentiality, including conducting interviews and workshops in private settings, securing data on password protected devices, keeping paper forms and tape recordings in locked cabinets/rooms, and maintaining all information collected by the study as confidential and not to be shared with individuals who are not part of the research team.

11.2 Benefits

Participants in this study will have convenient access to COVID-19 testing following an exposure. Household contacts of these participants will also have access to free COVID-19 self-tests should they wish to use them. There is no direct benefit to the community, however there may be indirect benefits by identifying more positive cases, which may reduce the spread of COVID-19 in the community.

This research will also be advantageous for academic study and in the future for other situations where serial ST may be helpful in identifying a contagious disease.

11.3 Study costs and compensation

No monetary compensation will be provided to participants for inclusion in the study. If any costs related to study participation are incurred by participants, they will be reimbursed by the study. No participant will be required to travel as part of their participation in this study.

11.4 COVID-19 considerations

Prior to study start, the study team will develop a Plan for Initiating Research that will address how study activities will be carried out as safely as possible, including the specific risk-reduction measures that will be taken to reduce risk of SARS-CoV-2 transmission and to ensure the safety of study participants, staff, and the community. This plan will be timely and tailored to be responsive to the COVID-19 epidemiological context at the study site at the time of study start. The study will adhere to all COVID-19-related guidance and requirements issued by national public health authorities, local governments, overseeing IRBs, Brazilian CEP/CONEP System, regulatory bodies, and study partners (PATH, CEPEM, and the Curitiba site), and other relevant entities as applicable.

11.5 Ethical Review

The protocol, informed consent form, and recruitment materials will be submitted to WHO Ethics Review Committee, CEP-CEPEM (CEPEM’s Research Ethical Committee,) the Curitiba site’s research ethical committee, and to CONEP (The National Commission for Research Ethics), for written approval.

All amendments and modifications will be submitted to the IRBs listed in this protocol for review and approval. No changes in protocol conduct will be implemented until approvals by all IRBs are obtained.

11.5.1 Managing and reporting adverse events and unanticipated problems

Any adverse events that are unanticipated, serious, and related or possibly related to participation in the research, any serious adverse events, or any incidents that suggest that the research places participants or others at risk, including breach of confidentiality will be promptly reported to the appropriate IRBs in accordance with the reporting requirements and required timeframe of all overseeing IRBs. A complete written report will follow the initial notification. Other incidents will be reported in the annual continuing review report.

11.5.2 Managing and reporting protocol deviations

Any deviation from the protocol that may have an impact on the safety or rights of the participant, or the integrity of the study will be promptly reported to the appropriate IRBs within the required timeframe from which the deviation is identified. All other deviations will be similarly reported to the appropriate IRBs in the annual continuing review report.

11.6 Compliance with Resolutions CNS 466/2012 and 580/2018

This study will comply with all Brazilian regulations regarding research with human subjects. The conduct of this study may affect healthcare workers' routines, as they may not be involved in any contact tracing at the time of protocol implementation, though may become involved in the study at some point during protocol execution. If the execution of this study impacts the ability of the healthcare system to function or increases the workload of healthcare workers, the study team will provide direct resources to carry out the necessary research activities.

11.7 Sex and Gender

This study is expected to generate knowledge applicable to men and women. Data collection will be desegregated by sex which would provide better insights on how to best implement self-testing for both sexes. Notably, the severity of the pandemic in Brazil has had documented and disproportionate impacts on women's equality (see https://www.genderandcovid-19.org/update/gender-and-covid-19-in-brazil-social-impacts-on-womens-lives/). This study aims to test a viable public health strategy that may serve to help limit new infections and mitigate some of the negative impacts.

Women may be more likely to seek information and care for COVID-19 for themselves or for family members and children they care for. This will be captured and documented through the follow-up surveys. Test instructions for use and training requirement needs may differ between different sexes and different genders. Recruitment of participants into the usability workshops and qualitative data collection will be balanced to include equal representations.

The study employs a passive recruitment strategy based on the incidence of COVID-19 infections at the study sites. Such strategy could in theory result in a less representative sample in terms of sex and gender. However, rates of covid-related infection and care-seeking are not significantly different between sexes and genders at the study sites and this is not expected.

The reporting of research findings will consider sex differences to ensure generalizability of the research findings and their applicability to both women and men. The studies will stratify the results based on sex and in particular:

- The terms "sex and gender" will be used in such a way as to avoid confusion and will be clearly distinguished.
- The final report of the study will include, where relevant, reporting of sex differences.

11.8 Special Considerations for Enrolling Minors

The consent/assent process for this study is tailored to three different age groups that may be enrolled in this study, per CONEP regulations. For the youngest age group assenting (7-11 years of age), the caregiver (parent/legal guardian giving consent) will perform the self-test on the

child. The other two age groups (12-14 and 15-17 years of age) will perform the self-test on themselves, though they may receive help from their caregiver as needed. This determination is consistent with what would reasonably be expected of these age groups and CONEP regulations. Additionally, the self-tests being used in this study are indicated for children 2 years and older with adult supervision, so all use during this study will be on-label.

Enrolling children also does not sufficiently alter the risk-benefit ratio to warrant excluding children from this study. The only additional risk this study poses to children is that they may be more likely to use the test incorrectly. This risk will be mitigated by conducting the test with caregiver supervision. Children may also benefit from the findings of this research given their participation in school and social group activities; they have also been disproportionately affected by the pandemic due to school closures. Self-testing is a strategy that can be used by individuals and families. This study will show how serial self-testing can be used in families and whether it can be operationalized to be effective. As such, it would be unethical to exclude children from this study, given their potential to benefit from the findings and minimal additional risk posed to them.

12.0 Study limitations

As with all research studies and particularly for COVID-19 research studies, this study has limitations. Study activities will be impacted by changing public health policies and guidelines as well as the ever-evolving epidemiology of COVID-19. Where possible, these will be monitored during the course of the study and tracked both administratively and through participant surveys. The methods outlined in this protocol are designed to be flexible enough to adapt to the local COVID-19 situation as needed while maintaining sufficient scientific rigor to fulfill our objectives. Additionally, follow-up data may be biased due to both the nature of being self-reported as well as observed (Hawthorne effect). Some of this bias, particularly related to isolation behaviors, will be mitigated through the use of GPS data. Data from secondary exposures may also be influenced by the fact their household member is participating in a research study. We feel this trade-off is appropriate considering the pragmatic study design we have chosen. Finally, we acknowledge that performing daily self-tests for 10 days is not a cost-effective method of contact tracing, rather we have chosen this timeframe and approach in the interest of generating a robust data set. Commodity costs will be tracked so that different testing regimens may be considered in the future.

In the event a participant is illiterate, an impartial witness will observe the consent process and attest that the information was explained as written and without undue influence (see section 7.1). These participants may elect to have a member of their household read the daily survey questions/other study materials to them or to have a member of the study team call them to complete study activities. Due to the study design (i.e., we must be able to reach close contacts to even enroll them in the study), it will not be possible for close contacts to participate in the study if they do not have access to telephone or electronic means of communication. Exceptions to this apply to index cases and secondary exposure contacts; if an index case does not have access to telephone or electronic means of communication, they will not be able to complete the daily surveys and we simply will be missing those data. The same can be said for secondary exposures, if they do not have access to electronic or telephone means of communications, they

will not be able to report any self-test use and we will simply miss those data. Given the extremely high coverage of phones in both Rondônia and Curitiba, we anticipate the incidence of missing data due to lack of electronic or telephone means of communication to be very low.

13.0 Research dissemination and stakeholder engagement

Stakeholders from the local municipalities and the Ministry of Health will be engaged during protocol development to obtain study buy-in. These channels of communication will remain open throughout the conduct of the study to ensure continued engagement, and results will be shared back with these stakeholders. In addition to local results sharing, study findings will be disseminated through a variety of channels, including the broader project consortium with Population Services International, the World Health Organization, and peer-reviewed publications. Research results will also be shared locally, at the participating facilities and health service units through debrief meetings and short reports. At the close of their participation, participants will also be sent information about where they can find final study results and be notified of dissemination outputs and events.

14.0 Investigator responsibilities and implementation

The three project partners involved in this research are PATH (prime award recipient and study sponsor), CEPEM, and the Curitiba site (CEPEM and Curitiba are implementing research partners). Roles and responsibilities for each of the partners are listed in the Table 5 below. (L= lead; A=assist).

Table 5: Investigator Responsibilities

Task	PATH	CEPEM/ ICC
Award oversight	L	A
Study design and protocol development	L	A
Develop data collection forms and study database	L	A
IRB submission: PATH/WIRB	L	A
Local IRB submissions	A	L
Study logistics arrangements	A	L
Procurement of self-tests and non-local study supplies	L	A
Procurement of all locally available study supplies	A	L
Implement study according to IRB-approved protocol	A	L
Data entry and cleaning	A	L
Data analysis and reporting	L	A
Dissemination	L	A

The study will be implemented after all necessary approvals are received. Study staff will be trained prior to implementation and recruitment will continue until a sufficient sample is reached. We anticipate 6 months will be needed to achieve the stated sample size, in addition to preparatory to research activities. Table 6 below describes the study timeline, and Table 7 describes the budget.

The investigator responsibilities are as follows:

- Dhélio Pereira, MD PhD will serve as the study principle investigator.
- Camilo Manchola-Castillo, PhD, will serve as technical advisor and the liaison between the research teams, national and municipal officials
- Alexandre Tavares Dias Costa, PhD will serve as the local principal investigator for the Curitiba site.
- Michelle Oliveira Silva, PhD will serve as the site coordinator for the Porto Velho site.
- Emily Gerth-Guyette and Rebecca Green support the design of the study and will support study initiation as well as data analysis.
- Gonzalo Domingo, Stephanie Zobrist, Kim Green and Phillips Loh will serve as key technical advisors.

Table 6: Study Timeline

Activity	*Month*									
	**1**	**2**	**3**	**4**	**5**	**6**	**7**	**8**	**9**	**10**
Ethical Approvals	X	X								
Protocol Training and Kick-Off			X							
Procurement of Study Supplies	X	X	X							
Recruitment				X	X	X	X	X	X	
Data Cleaning and Analysis									X	X
Report Development and Dissemination										X

References:

1. Johns Hopkins University. https://coronavirus.jhu.edu/data/new-cases.

2. Lipsitch M, Swerdlow DL, Finelli L. Defining the Epidemiology of Covid-19 — Studies Needed. *N Engl J Med*. 2020;382(13):1194-1196. doi:10.1056/NEJMp2002125

3. WHO COVID-19 Case definition. Accessed February 2, 2022. https://www.who.int/publications-detail-redirect/WHO-2019-nCoV-Surveillance_Case_Definition-2020.2

4. Tang YW, Schmitz JE, Persing DH, Stratton CW. Laboratory Diagnosis of COVID-19: Current Issues and Challenges. *J Clin Microbiol*. 2020;58(6):e00512-20. doi:10.1128/JCM.00512-20

5. Peeling RW, Olliaro P. Rolling out COVID-19 antigen rapid diagnostic tests: the time is now. *Lancet Infect Dis*. 2021;21(8):1052-1053. doi:10.1016/S1473-3099(21)00152-3

6. Bekliz M, Perez-Rodriguez F, Puhach O, et al. Sensitivity of SARS-CoV-2 antigen-detecting rapid tests for Omicron variant. Published online January 17, 2022:2021.12.18.21268018. doi:10.1101/2021.12.18.21268018

7. Krüger LJ, Gaeddert M, Tobian F, et al. The Abbott PanBio WHO emergency use listed, rapid, antigen-detecting point-of-care diagnostic test for SARS-CoV-2—Evaluation of the accuracy and ease-of-use. *PLOS ONE*. 2021;16(5):e0247918. doi:10.1371/journal.pone.0247918

8. Brümmer LE, Katzenschlager S, Gaeddert M, et al. Accuracy of novel antigen rapid diagnostics for SARS-CoV-2: A living systematic review and meta-analysis. *PLOS Med*. 2021;18(8):e1003735. doi:10.1371/journal.pmed.1003735

9. Batista C, Hotez P, Amor YB, et al. The silent and dangerous inequity around access to COVID-19 testing: A call to action. *eClinicalMedicine*. 2022;43:101230. doi:10.1016/j.eclinm.2021.101230

10. Hodges E, Lefferts B, Bates E, et al. Use of Rapid Antigen Testing for SARS-CoV-2 in Remote Communities - Yukon-Kuskokwim Delta Region, Alaska, September 15, 2020-March 1, 2021. *MMWR Morb Mortal Wkly Rep*. 2021;70(33):1120-1123. doi:10.15585/mmwr.mm7033a3

11. How accurate are rapid tests for diagnosing COVID-19? doi:10.1002/14651858.CD013705.pub2

12. Goggolidou P, Hodges-Mameletzis I, Purewal S, Karakoula A, Warr T. Self-Testing as an Invaluable Tool in Fighting the COVID-19 Pandemic. *J Prim Care Community Health*. 2021;12:21501327211047784. doi:10.1177/21501327211047782

13. Wanat M, Logan M, Hirst JA, et al. Perceptions on undertaking regular asymptomatic self-testing for COVID-19 using lateral flow tests: a qualitative study of university students and staff. *BMJ Open*. 2021;11(9):e053850. doi:10.1136/bmjopen-2021-053850

14. Shilton S, Ivanova Reipold E, Roca Álvarez A, Martínez-Pérez GZ. Assessing Values and Preferences Toward SARS-CoV-2 Self-testing Among the General Population and Their Representatives, Health Care Personnel, and Decision-Makers: Protocol for a Multicountry Mixed Methods Study. *JMIR Res Protoc*. 2021;10(11):e33088. doi:10.2196/33088

15. Sibanda E, Choko AT, Watadzaushe C, et al. Use of SARS-CoV-2 antigen-detection rapid diagnostic tests for COVID-19 self-testing: interim guidance, 9 March 2022: web annex B: COVID-19 self-testing using antigen rapid diagnostic tests: feasibility evaluation among health-care workers and general population in Malawi and Zimbabwe. Published online 2022. Accessed May 10, 2022. https://policycommons.net/artifacts/2270225/use-of-sars-cov-2-antigen-detection-rapid-diagnostic-tests-for-covid-19-self-testing/3030039/

16. Pray IW. Performance of an Antigen-Based Test for Asymptomatic and Symptomatic SARS-CoV-2 Testing at Two University Campuses — Wisconsin, September–October 2020. *MMWR Morb Mortal Wkly Rep*. 2021;69. doi:10.15585/mmwr.mm695152a3

17. Juneau CE, Pueyo T, Bell M, Gee G, Collazzo P, Potvin L. *Evidence-Based, Cost-Effective Interventions To Suppress The COVID-19 Pandemic: A Systematic Review*. Public and Global Health; 2020. doi:10.1101/2020.04.20.20054726

18. Juneau CE, Briand AS, Pueyo T, Collazzo P, Potvin L. Effective Contact Tracing for COVID-19: A Systematic Review. Published online July 25, 2020:2020.07.23.20160234. doi:10.1101/2020.07.23.20160234

19. Guia de Vigilância Epidemiológica Covid-19: Emergência da Saúde Pública de Importância Nacional pela Doença pelo Coronavírus 2019 - Covid-19 — Português (Brasil). Accessed September 1, 2022. https://www.gov.br/saude/pt-br/centrais-de-conteudo/publicacoes/publicacoes-svs/coronavirus/guia-de-vigilancia-epidemiologica-covid-19_2021.pdf/view

20. Cartilha - Plano Nacional de Expansão da Testagem para Covid-19 (PNE-Teste) — Português (Brasil). Accessed September 1, 2022. https://www.gov.br/saude/pt-

br/centrais-de-conteudo/publicacoes/cartilhas/2021/plano-nacional-de-expansao-da-testagem-para-covid-19.pdf/view

21. Cheng HY, Jian SW, Liu DP, et al. Contact Tracing Assessment of COVID-19 Transmission Dynamics in Taiwan and Risk at Different Exposure Periods Before and After Symptom Onset. *JAMA Intern Med*. 2020;180(9):1156-1163. doi:10.1001/jamainternmed.2020.2020

22. Brazilian regulators approve self testing kits. https://brazilian.report/liveblog/2022/01/28/approve-covid-self-testing-kits/ or the tools offered on the page.

23. Brazillian Ministry of Health. *Epidemiological Surveillance Guide*.; 2022.

24. Recommendations for Isolation of COVID-19 Cases. Presented at: January 10, 2022.

25. Zobrist S, Oliveira-Silva M, Vieira AM, et al. Screening for SARS-CoV-2 in close contacts of individuals with confirmed infection: performance and operational considerations. Published online January 28, 2022:2022.01.27.22269904. doi:10.1101/2022.01.27.22269904

26. Virzi RA. Refining the Test Phase of Usability Evaluation: How Many Subjects Is Enough? *Hum Factors*. 1992;34(4):457-468. doi:10.1177/001872089203400407

27. Damschroder LJ, Aron DC, Keith RE, Kirsh SR, Alexander JA, Lowery JC. Fostering implementation of health services research findings into practice: a consolidated framework for advancing implementation science. *Implement Sci IS*. 2009;4:50. doi:10.1186/1748-5908-4-50
