# Supplementary material for: Integration of serial self-testing for COVID-19 as part of contact tracing in the Brazilian public health system: A pragmatic trial protocol
Source: PLoS One. 2023 Oct 4;18(10):e0284659. doi: 10.1371/journal.pone.0284659 (PMC10550143; doi:10.1371/journal.pone.0284659)
Supplement: S2 File — (PDF) [file pone.0284659.s002.pdf]

Informed Consent Form: CLOSE CONTACTS (ADULT – 18 years or older)

Study title: A Pragmatic Trial to Integrate Serial Self-Testing into Public Health Contact Tracing Programs: A Study to Assess the Operational Feasibility and Impact of Self-Testing among Exposed Individuals

**Principal investigator:**	Dhélio Pereira, MD PhD<br>Clinical Director, Centro de Pesquisa em Medicina Tropical de Rondônia
**Co-investigator:**	Alexandre Dias T. Costa, MD, PhD Alexandre Dias T. Costa, MD, PhD<br>Especialista em Saúde Pública, Fiocruz Paraná
**Study conducted by:**	Centro de Pesquisa em Medicina Tropical de Rondônia (CEPEM) and Ouvidor Pardinho Health Unit/Fiocruz-Paraná
**Sponsor:**	PATH

INFORMATION ABOUT THIS STUDY

The Centro de Pesquisa em Medicina Tropical de Rondônia (CEPEM), Ouvidor Pardinho Health Unit, and PATH are working on a study to learn more about how people can use coronavirus (COVID-19) diagnostic tests. PATH is an international public health organization.

COVID-19 is an infectious disease caused by a virus called SARS-CoV-2. Some people with the virus can become sick, and some people have no symptoms. Some symptoms of COVID-19 are similar to the flu, for example: fever, chills, sore throat, headache, cough. A person who has the virus can give it to other people, even if they have no symptoms. Testing for COVID-19 is important to know if someone has the virus and can pass it to other people.

We are inviting you to participate in a research study. We invite you to join the study because you have come into contact with someone who has tested positive for COVID-19. In this study, we want to learn about a new way to test for COVID-19 that can be done at home. There are some new tests for COVID-19 that don't need to be done in a clinic. We want to see if these new tests work well. What we learn from this study can help us to improve the testing process for COVID-19 so more people will be able to access tests. We hope to enroll 604 people in this study who have had close contact with a person who has tested positive for COVID-19.

This consent form gives you information about the study. Use as much time as you need to understand what will happen, the risks, and the benefits. Feel free to ask us any questions. When we have answered all your questions, you can decide whether or not to participate. If you choose to participate, we will ask you to sign this form. You will receive a copy to keep. The form will be signed by you and a member of the study team.

WHAT WILL HAPPEN IN THE STUDY?

If you agree to participate and meet the requirements, the following will happen:

- You will sign this consent form, agreeing to be in the study.
- You will need to be with the study team for up to 45 minutes.
- We will ask you some information about yourself (age, sex, ethnicity), your health, and if you have any symptoms of COVID-19.
- We will give you a test for COVID-19 and instructions on how to do it yourself. You will know the results of this test within 30 minutes. Care will be offered if you test positive.

- We will swab your nose. This sample will be tested right away using a rapid test for COVID-19.
- We may give you more tests for COVID-19 to do at home over the next 10 days. We may also give you extra tests to give to people you live with.
- We will ask you to fill out a survey, either online or by calling us on the phone, every day for the next 10 days.

HOW DOES THIS STUDY DIFFER FROM STANDARD CARE?

In standard medical care, you would be seen and treated by a health professional. In this study, you will know your COVID-19 test result. You may also get tests to do at home. We will refer you for the treatment of symptoms and provide information that helps you obtain medical care in a public clinic or hospital. It is important to know that the treatment of COVID-19 is not the object of this study, only the new testing methods we are studying. The rapid tests we will give you today to take home is not part of the standard care. However, this test is approved for use in Brazil.

HOW WE WILL PROTECT YOUR PRIVACY

We will protect your privacy in this study. We will use a number instead of your name in the study forms (for example: 27613). We will keep the study forms locked and the computers protected with a password. During the study, we will use a key to link your name with the study number so we can share the results of your lab test with you. Only the study staff will have access to the key.

All suspected and confirmed positive cases of COVID-19 will be reported by CEPEM to the public health authorities in Brazil, as required by law. Groups that supervise or fund the study may have access to the results. This includes members of the ethics committees of PATH, the Ethics Committee from the Center for Research in Tropical Medicine (CEP / CEPEM), the National Commission for Research Ethics (CONEP) and other test regulators. These people agree to keep your information private. There will be no access to data that identifies you. No person or group will have access to your data without agreement from the study team. They will only have access under supervision.

We will use the information to assess tests that detect COVID-19. The information we collect will be shared with our funders. It may be made open to the public so others can learn from it. If the data is shared publicly, it will not be linked to you.

BENEFITS

The results of the study tests may be useful for your care. What we learn may help others in the future who require diagnostic testing for COVID-19.

COST

You will not be paid to participate in the study. You will not be charged for participating in the study. We will reimburse any costs spent by you, or your companion, because of your participation in the study. If you suffer any damage from participation in the study, the team responsible for the research will provide immediate and comprehensive assistance. If you suffer any damage resulting from your participation in the research, you have the right to seek for repair.

CAN SOMETHING BAD HAPPEN TO ME FOR PARTICIPATING IN THIS STUDY?

Swabbing your nose may be uncomfortable and could cause your nose to bleed. It is also possible the rapid test may give you an incorrect test result. There is less than a 5% chance this will happen. However, this chance might go up when taking multiple tests, which we are asking you to do in this study. If you think the test result is wrong, we will help you get a confirmatory test from a lab. There may be risks or side effects which are unknown at this time.

Another risk in this study is that unauthorized persons see your records by mistake. If this happens, you will be informed.

No injuries are expected in this study. However, if you are injured by sample collection, please let the study team know and they will treat you or refer you for treatment. The study will pay for any care you need. It is important to note that any injury is the responsibility of the investigators and will not cause expenses to your health plan, SUS, or even to you. All study procedures are the responsibility of the researchers, including all costs associated with the procedures. You do not give up any of your legal rights by signing this consent form.

VOLUNTARY PARTICIPATION:

Participation in this study is optional. You can say no. You may decide not to participate in this study or stop your participation at any time, even if you already agreed. It is not necessary to give a reason. If you decline or stop your participation in this study, there will be no negative consequences, penalty, or loss of benefits to which you are otherwise entitled. You will still be able to receive treatment at your local health center.

Your alternative to participating in the research is not to participate. The study may be stopped with or without prior approval from CEP, when necessary to protect you and other participants.

DOUBTS ABOUT THE STUDY?

You may be concerned or have questions about your participation in the study or your information. Please talk about your concerns, or complaints, with the study team at any time. If you have further questions about the research, or if you feel you have been injured, please contact Dr. Dhélio Pereira at (69) 3216-5442, or by email dbpfall@gmail.com. Dr. Dhelio Pereira’s address is Av. Guaporé nº 215, Bairro Lagoa.

If you have doubts about your rights as a research participant, or feel you have been harmed, contact your local Research Ethics Committee:

- Porto Velho: CEPEM Research Ethics Committee at (069) 3216 -5442, or by email at cepesquisacepem@yahoo.com.br. CEPEM’s CEP address is: Av. Guaporé nº 215, Bairro Lagoa (Anexo ao Hospital CEMETRON).
- Curitiba: Comitê de Ética em Pesquisa da Secretaria Municipal da Saúde (41) 3360-4961, or by email at etica@sms.curitiba.pr.gov.br. The CEP address is: Rua Atilio Bório, nº 680, Bairro Cristo Rei, Curitiba

You can also contact CONEP by phone (61) 3315-5877, from Monday to Friday from 9h to 18h and email conep@saude.gov.br. CONEP’s address is SRTV 701, Via W 5 Norte, lote D – Edifício PO 700, 3º andar – Asa Norte, Brasília, DF.

The CEP is a committee made up of people from different areas, whose main functions are to protect the research participants' interest in their integrity and dignity and to contribute to the development of research within ethical standards. The CEP can help if you feel harmed by the research team or if you have questions about ethical standards. The CEP is open Monday through Friday from 8:00 am to 2:00 pm.

VOLUNTEER'S DECLARATION

The research study was explained to me. I had the opportunity to ask questions. All my questions have been answered. If I have further questions about the study, I can ask a member of the study team who will respond or give me a location where I can get the answers. I will receive a copy of this consent form.

I will voluntarily participate in the research study.

CONSENT SIGNATURE

____
Participant's name (print)

____ __________________
Participant's signature (required if 18 years or older) Date

Study Team:
By signing below, I confirm that I explained this study to you and that I answered your questions to the best of my knowledge. I will give you a signed copy of this document for you to keep.

____
Name of study team member who explained the consent form (in full, print)

____
Signature of study team member who obtained consent

____
Date

FOR PARTICIPANTS WHO CANNOT READ OR WRITE:

VOLUNTEER'S DECLARATION

The research study was explained to me. I had the opportunity to ask questions. All my questions have been answered. If I have further questions about the study, I can ask a member of the study team who will respond or give me a location where I can get the answers. I will receive a copy of this consent form.

I will voluntarily participate in the research study.

CONSENT SIGNATURE

____
Participant's name (to be completed by person obtaining consent)

____
Date (to be completed by person obtaining consent)

Participant thumbprint

Study Team:
By signing below, I confirm that I explained this study to you and that I answered your questions to the best of my knowledge. I will give you a signed copy of this document for you to keep.

____
Name of study team member who explained the consent form (in full, print)

____ ________________
Signature of study team member who obtained consent Date

Witness:
By signing below, I confirm that the study participant, whose fingerprint is above, chose to participate in this research study. I also confirm that I was present during the whole time the study was explained. I also confirm that the participant had the opportunity to ask questions. The participant will receive a copy of this form to keep.

____
Full name of witness (print)

____ ________________________
Witness signature Date
